# Dynamics and Persistence of a Generalized Multi-strain SIS Model

**DOI:** 10.1007/s11538-025-01516-z

**Published:** 2025-09-10

**Authors:** Scott Greenhalgh, Tabitha Henriquez, Michael Frutschy, Rebecah Leonard

**Affiliations:** 1 Department of Mathematics, Siena University, 515 Loudon Road, Loudonville, NY 12211 USA; 2 Data Science Program, Siena University, 515 Loudon Road, Loudonville, NY 12211 USA

**Keywords:** Stability analysis, Floquet theory, Ordinary differential equations, Syphilis, Akaike information criteria, Forecast skill score

## Abstract

Autonomous differential equation compartmental models hold broad utility in epidemiology and public health. However, these models typically cannot account explicitly for myriad factors that affect the trajectory of infectious diseases, with seasonal variations in host behavior and environmental conditions as noteworthy examples. Fortunately, using non-autonomous differential equation compartmental models can mitigate some of these deficiencies, as the inclusion of time-varying parameters can account for temporally varying factors. The inclusion of these temporally varying factors does come at a cost though, as many analysis techniques, such as the use of Poincaré maps and Floquet theory, on non-autonomous differential equation compartmental models are typically only tractable numerically. Here, we illustrate a rare $n$-strain generalized Susceptible-Infectious-Susceptible (SIS) compartmental model, with a general time-varying recovery rate, which features Floquet exponents that are algebraic expressions. We completely characterize the persistence and stability properties of our $n$-strain generalized SIS model for $n\ge 1$. We also derive a closed-form solution in terms of elementary functions for the single-strain SIS model, which is capable of incorporating almost any infectious period distribution. Finally, to demonstrate the applicability of our work, we apply it to recent syphilis incidence data from the United States, utilizing Akaike Information Criteria and Forecast Skill Scores to inform on the model’s goodness of fit relative to complexity and the model’s capacity to predict future trends.

**Supplementary Information:**

The online version contains supplementary material available at 10.1007/s11538-025-01516-z.

## Introduction

Many infectious disease trajectories are critically affected by seasonal effects. The exact causes of these seasonal effects are often intensely contested (Feng and Thieme 1995), with changes in host behavior, such as contact rate, or environmental conditions, such as humidity (Fares 2013), frequently regarded as leading covariates. In this regard, considerable work has been done on how these covariates may affect infectious disease trajectories through differential equation compartmental models (Greenhalgh and Rozins 2021). Often, these compartmental models are of the autonomous type and are designed to describe the particular epidemiology of a disease. By creating autonomous compartmental models to describe diseases, one can then apply a plethora of analysis techniques to inform on disease dynamics, including the use of stability analysis and Hopf bifurcation theory to inform on long-term solution behavior (Greenhalgh 1997; Greenhalgh et al. 2015a; Al-arydah et al. 2020) or a combination of health-benefit analysis, cost-effectiveness analysis (Ghani et al. 2009; Mbah et al. 2014; Greenhalgh et al. 2015b, 2018; Brock et al. 2017; Greenhalgh and Chandwani 2020), and optimal control theory (Jung et al. 2002; Joshi et al. 2006; Mallela et al. 2016; Bonyah et al. 2019; Kouidere et al. 2020), among others, to inform on the merits of novel disease interventions and public health policies.

Non-autonomous compartmental models can also be applied in a similar fashion to their autonomous counterparts. Still, many infectious disease modelers often underutilize them due to the complexities that arise from time-varying parameters. Case in point, the typical procedure for the stability analysis of non-autonomous compartmental models requires some form of Poincaré map, time-averaging (Greenhalgh and Moneim 2003; Moneim and Greenhalgh 2005; Ma and Ma 2006), which does not always hold (Wang and Zhao 2008), or the application of Floquet theory, to inform on long-term solution behavior. Further exacerbating these complexities is that many of these approaches can only be done numerically (LUST 2001; Kuznetsov 2004), which is likely partially why some novice disease modelers avoid their use. While there are experts who elegantly apply these analyses to non-autonomous compartmental models (Feng and Thieme 1995; Martcheva 2009; Tian and Wang 2015; Nill 2023), even the stability of multi-strain periodic solutions of the non-autonomous Susceptible-Infectious-Susceptible (SIS) model with more than two strains remains an open question (Martcheva 2009).

The SIS model and its extensions are amongst the most well-studied of all compartmental models. With respect to the SIS model, it has many convenient properties, including an equivalent reformulation as the logistic growth equation, a closed-form solution in terms of elementary function (Brauer 2008), disease-free equilibrium (DFE), endemic equilibrium, and simple stability criteria, which are easily derived from Jacobian, next-generation, and other methods (Otto and Day 2007; Martcheva 2015; Lloyd 2017; Blackwood and Childs 2018). The extensions of SIS models often share many of these properties, with numerous works developing criteria for strains to exclude one another or permit co-existence [32, 33], studies that address the effects of periodic parameters (Martcheva 2009) for up to two strains, along with works that extend the closed-form solution of SIS models to ones that permit periodic behavior (Greenhalgh and Dumas 2023). However, existing models do not fully address the complex dynamics of $n$-strain SIS models. So, we address this gap by developing a novel multi-strain SIS model, obtaining its extinction and persistence criteria for single and multi-strain periodic solutions.

To illustrate our work, we utilize Center for Disease Control and Prevention (CDC) data on syphilis incidence in the US. Typically, the transmission route of syphilis is through sexual contact, although other transmission routes are possible, such as mother-to-child transmission. While mother-to-child transmission is rare, syphilis annually infects over 207,000 people in the US (Center for Disease Control and Prevention 2024), which represents a fivefold increase as of 2019 (Amerson et al. 2022). Because of this, syphilis poses a considerable public health challenge (Levine 2024), in part because it is a sexually transmitted bacterial infection that does not confer definitive and protective immunity against reinfection (Marchese et al. 2022). Given these infection characteristics, syphilis dynamics should mirror an SIS model. So, we apply our generalized SIS model (gSIS) and the traditional SIS model to describe its trends and contextualize our results.

## Methods

We generalized an SIS model to track the quantity of person-days of infection throughout a population. To place our work in context, we overview single-strain SIS and gSIS models, their formulations with $n$-strains, and illustrate their basic solution properties.

### The Traditional SIS Model

#### The Single-Strain SIS Model

For the single-strain SIS model, we denote susceptible individuals as $S$, and infected individuals as $I$. We assume the transition between $S$ and $I$ is governed by the differential equation system,

1 $$\begin{gathered} S^{\prime} = - \frac{\beta }{N}SI + \gamma I, \hfill \\ I^{\prime} = \frac{\beta }{N}SI - \gamma I. \hfill \\ \end{gathered}$$

Here, $\beta$ is the transmission rate, $N$ is the total population, and $\gamma$ is the recovery rate.

The traditional SIS model has two equilibrium solutions. The first is the DFE,

$$\hat{S} = N,\hat{I} = 0,$$

and the second is the endemic equilibrium,

$$\tilde{S} = N\frac{\gamma }{\beta }, \tilde{I} = N\left( {1 - \frac{\gamma }{\beta }} \right).$$

The SIS model is also a rare differential equation compartmental model with a closed-form solution (Martcheva 2015), as when $\beta \ne \gamma ,$

2 $$\begin{array} {c}S = N - I, {\text{ and }} I = \frac{{N\left( {\beta - \gamma } \right)I_{0} }}{{\beta I_{0} + \left( {N\left( {\beta - \gamma } \right) - \beta I_{0} } \right)e^{{ - \left( {\beta - \gamma } \right)t}} }},  {\text{ and for }}\;\beta = \gamma , S = N - I, {\text{ and }} I = \frac{{I_{0} }}{{1 + \gamma \;\frac{{I_{0} }}{N}t}}. \end{array}$$

#### The ${\varvec{n}}$-Strain SIS Model

The SIS model can be extended into an $n$-strain system. Denoting ${I}_{j}$ as the strain $j$ infected individuals, the $n$-strain SIS model is given by

$$\frac{dS}{{dt}} = \mathop \sum \limits_{j = 1}^{n} - \frac{{\beta_{j} }}{N}I_{j} S + \gamma_{j} I_{j} ,$$

$$\frac{{dI_{j} }}{dt} = \frac{{\beta_{j} }}{N}I_{j} S - \gamma_{j} I_{j} , \forall j \in \left\{ {1 \ldots n} \right\}.$$

Naturally, one can solve the right-hand side of the system set equal to zero to determine the DFE,

3 $$S = N, I_{j} = 0, \forall j$$

or single-strain endemic equilibria,

4 $$S = N\frac{{\gamma_{j} }}{{\beta_{j} }}, I_{j} = N\left( {1 - \frac{{\gamma_{j} }}{{\beta_{j} }}} \right), I_{k} = 0, \forall k \ne j.$$

Under the condition where $\frac{{\beta }_{k}}{{\gamma }_{k}}=\underset{j}{\text{max}}\frac{{\beta }_{j}}{{\gamma }_{j}}>1$ for $k\in\Omega \subseteq \left\{1\dots n\right\},$ a co-existence endemic equilibrium exists,

5 $$S = N\frac{{\gamma_{j} }}{{\beta_{j} }}, I_{j} = \omega_{j} N\left( {1 - \frac{{\gamma_{j} }}{{\beta_{j} }}} \right), \forall j \in {\Omega },{\text{and}} I_{l} = 0, {\text{for }}l \in \left\{ {1, \ldots ,n} \right\}\backslash {\Omega },$$

where each $\omega_{j}$ is determined through

6 $$\mathop \sum \limits_{{i \in {\Omega }}} \tilde{I}_{i} = N\left( {1 - \frac{{\gamma_{j} }}{{\beta_{j} }}} \right) {\text{for any }}j \in {\Omega },$$

and through the integration of

7 $$\frac{1}{{\beta_{j} I_{j} }}\frac{{dI_{j} }}{dt} - \frac{1}{{\beta_{k} I_{k} }}\frac{{dI_{k} }}{dt} = - \frac{{\gamma_{j} }}{{\beta_{j} }} + \frac{{\gamma_{k} }}{{\beta_{k} }},$$

for $j,k \in \left\{ {1, \ldots ,n} \right\},$ which yields

8 $$\frac{1}{{\beta_{j} }}\left( {\ln I_{j} - \ln I_{j} \left( 0 \right)} \right) - \frac{1}{{\beta_{k} }}\left( {\ln I_{k} - \ln I_{k} \left( 0 \right)} \right) = - \left( {\frac{{\gamma_{j} }}{{\beta_{j} }} - \frac{{\gamma_{k} }}{{\beta_{k} }}} \right)t = 0.$$

Specifically, using (6) and (8), one obtains an implicit equation for the endemic equilibrium value of each ${\widetilde{I}}_{j}$ in terms of its initial conditions and parameters.

### A Generalized SIS Model

**Fig. 1:**
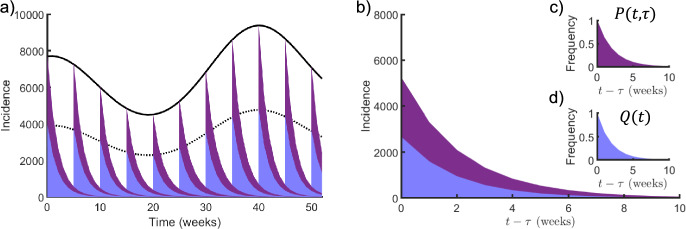
The survival of infected individuals throughout an outbreak. **a** The trajectory of incidence (solid black curve), new incidence (dotted black curve), and the survival of newly infected individuals for remaining infectious at 5-week intervals (blue regions), and the survival of all previously infected individuals for remaining infectious at 5-week intervals (purple plus blue regions) over a total of 50 weeks. **b** The survival of newly infected individuals for remaining infectious (blue regions), and the survival of all previously infected individuals for remaining infectious (purple plus blue regions) for a given initial time $\tau$, **c** the survival function of the duration of infection, $P(t,\tau )$, and **d** the survival function of the infectious period, $Q(t)$. Reproduced with permission from (Greenhalgh and Dumas 2023) (Color figure online)

We characterize the spread of disease throughout a population using the quantity of person-days of infection rather than incidence (Greenhalgh and Rozins 2021). The quantity of person-days of infection amounts to the area enclosed by a survival curve for remaining infected multiplied by the number of individuals infected with a disease at a particular moment throughout an epidemic (Fig. 1). This area includes both new infections, ${I}_{new}(t)$, which decay according to the infectious period distribution, and prior infections, which decay according to a conditional infectious period distribution (Fig. 1), and amount to the product of the currently infected individuals by their (time-varying) average duration of infection, $m\left(t\right)$*,* or more specifically, a mean residual waiting-time (Greenhalgh and Dumas 2023). Formally, we consider person-days of infection as,

$$\mathop \smallint \limits_{{\text{x}}}^{\infty } I\left( x \right)P\left( {t,x} \right)dt = I\left( x \right)m\left( x \right),$$

where $P\left(t,x\right)=\text{exp}\left(-{\int }_{x}^{t}\frac{{m}^{\prime}+1}{m}dz\right)$ is the conditional survival function of the duration of infection distribution, given time $x$.

We can track the evolution of person-days of infection by the integral equations (Greenhalgh and Rozins 2021),

9 $$\begin{gathered} m\left( t \right)S\left( t \right) = m\left( t \right)N - m_{0} I_{0} Q\left( t \right) - \mathop \smallint \limits_{0}^{t} m_{0} I_{new} \left( x \right)Q\left( {t - x} \right)dx, \hfill \\ m\left( t \right)I\left( t \right) = m_{0} I_{new} \left( 0 \right)Q\left( t \right) + \mathop \smallint \limits_{0}^{t} m_{0} I_{new} \left( x \right)Q\left( {t - x} \right)dx, \hfill \\ \end{gathered}$$

where $m\left(0\right)={m}_{0}$ and $Q\left(t\right)$ is the survival function of the infectious period.

Typically, standard approaches reduce integral equations to differential equation formulations by invoking the law of mass action and replacing the infectious period distribution with the duration of infection distribution (Kermack and McKendrick 1991a, b, c). For (9), this is akin to the assumption,

$$m_{0} I_{new} \left( x \right)Q\left( {t - x} \right) = \frac{\beta }{N}I\left( x \right)S\left( x \right)m\left( x \right)P\left( {t,x} \right).$$

Under such modifications, we reduce (9) to the system of differential equations given by,

10 $$\begin{gathered} \left( {mS} \right)^{\prime} = Nm^{\prime} - \frac{\beta }{N}SIm + \frac{{m^{\prime} + 1}}{m}Im, \hfill \\ \left( {mI} \right)^{\prime} = \frac{\beta }{N}SIm - \frac{{m^{\prime} + 1}}{m}Im. \hfill \\ \end{gathered}$$

#### The Single-Strain gSIS Model

In (10), isolating for ${S}^{\prime}$ and ${I}^{\prime}$, we can equivalently rewrite the system as

11 $$\begin{gathered} S^{\prime} = N\frac{{m^{\prime}}}{m} - \frac{\beta }{N}SI + \frac{{m^{\prime} + 1}}{m}I - \frac{{m^{\prime}}}{m}S, \hfill \\ I^{\prime} = \frac{\beta }{N}SI - \frac{{2m^{\prime} + 1}}{m}I. \hfill \\ \end{gathered}$$

The difference between the single-strain SIS and gSIS models ultimately is the inclusion of a time-varying recovery term, $\frac{2{m}^{\prime}+1}{m}I,$ instead of one with a constant recovery rate, $\gamma I$. Note, this implies that our gSIS model is merely an SIS model with a time-varying recovery rate.

Under the assumption that $m$ approaches a constant value sufficiently fast, the single-strain gSIS has two equilibrium solutions. The DFE is given by

12 $$\hat{S} = N, \hat{I} = 0,$$

and the endemic equilibrium,

13 $$\tilde{S} = N\frac{1}{{\beta \tilde{m}}}, \tilde{I} = N\left( {1 - \frac{1}{{\beta \tilde{m}}}} \right),$$

where $$\underset{t\to \infty }{\text{lim}}m=\widetilde{m}=\text{constant}.$$

The behavior of (11) under different conditions on $m$ may not have an endemic equilibrium and could converge to a periodic trajectory. To illustrate this, we first note that ${I}^{\prime}$ is a Bernoulli differential equation (Buckley 1953) and has the solution

14 $$\begin{gathered} S = N - I, \hfill {\text{and}} \hfill I = \frac{{I_{0} \mu \left( t \right)}}{{1 + \beta \frac{{I_{0} }}{N}\mathop \smallint \nolimits_{0}^{t} \mu \left( z \right)dz}} = \frac{N}{\beta }\frac{d}{dt}\ln \left( {1 + \beta \frac{{I_{0} }}{N}\mathop \smallint \limits_{0}^{t} \mu \left( z \right)dz} \right). \hfill \\ \end{gathered}$$

For $I$ to have a closed-form solution, we require that $\mathop \smallint \limits_{0}^{t} \mu \left( z \right)dz$ can be expressed in terms of elementary functions. One case occurs by imposing

15 $$m = p\left( t \right)e^{\rho t} ,$$

and thus

16 $$\frac{{2m^{\prime} + 1}}{m} = \frac{{2p^{\prime}}}{p} + 2\rho + \frac{1}{p}e^{ - \rho t} = 2\rho - 2\frac{{f_{k}^{\prime} \left( t \right)}}{{f_{k} \left( t \right)}},$$

where

17 $$f_{k} \left( t \right) = \frac{{a_{0} }}{2} + \mathop \sum \limits_{j = 1}^{k} a_{j} \cos \left( {\frac{j\pi }{L}t} \right) + b_{j} \sin \left( {\frac{j\pi }{L}t} \right).$$

Solving $$p\left( t \right) = \frac{1}{{2f_{k} \left( t \right)}}\left( {\mathop \smallint \limits_{0}^{t} f_{k} \left( z \right)e^{ - \rho z} dz + 2p_{0} f_{k} \left( 0 \right)} \right)$$ yields

18 $$\begin{aligned} p\left( t \right) =\,& \frac{{e^{ - \rho t} }}{{2f_{k} \left( t \right)}}\left( \frac{1}{2}\frac{{a_{0} }}{\rho } + \mathop \sum \limits_{j = 1}^{k} \frac{{L\left( {\rho La_{j} + \pi jb_{j} } \right)}}{{\rho^{2} L^{2} + j^{2} \pi^{2} }}\cos \left( {\frac{j\pi t}{L}} \right)\right.\\ &\left. + \frac{{L\left( {\rho Lb_{j} - \pi ja_{j} } \right)}}{{\rho^{2} L^{2} + j^{2} \pi^{2} }}\sin \left( {\frac{j\pi t}{L}} \right) \right) \hfill \\ &- \frac{1}{{2f_{k} \left( t \right)}}\left( {\frac{1}{2}\frac{{a_{0} }}{\rho } + \mathop \sum \limits_{j = 1}^{k} \frac{{L\left( {\rho La_{j} + \pi jb_{j} } \right)}}{{\rho^{2} L^{2} + j^{2} \pi^{2} }}} \right) + p_{0} \frac{{f_{k} \left( 0 \right)}}{{f_{k} \left( t \right)}}. \hfill \\ \end{aligned}$$

For $m\left( t \right)$ to be periodic, we require

19 $$p_{0} = \frac{1}{{2f_{k} \left( 0 \right)}}\left( {\frac{1}{2}\frac{{a_{0} }}{\rho } + \mathop \sum \limits_{j = 1}^{k} \frac{{L\left( {\rho La_{j} + \pi jb_{j} } \right)}}{{\rho^{2} L^{2} + j^{2} \pi^{2} }}} \right).$$

Thus,

20 $$\begin{aligned} m\left( t \right) =\,& \frac{1}{{2f_{k} \left( t \right)}}\left( \frac{1}{2}\frac{{a_{0} }}{\rho } + \mathop \sum \limits_{j = 1}^{k} \frac{{L\left( {\rho La_{j} + \pi jb_{j} } \right)}}{{\rho^{2} L^{2} + j^{2} \pi^{2} }}\cos \left( {\frac{j\pi t}{L}} \right)\right.\\ &\left. + \frac{{L\left( {\rho Lb_{j} - \pi ja_{j} } \right)}}{{\rho^{2} L^{2} + j^{2} \pi^{2} }}\sin \left( {\frac{j\pi t}{L}} \right) \right).\end{aligned}$$

Under these assumptions, we have that

21 $$\mu \left( t \right) = \frac{{f_{k}^{2} \left( t \right)}}{{f_{k}^{2} \left( 0 \right)}}e^{{\left( {\beta - 2\rho } \right)t}}$$

and

22 $$\mathop \smallint \limits_{0}^{t} \mu \left( z \right)dz = \mathop \smallint \limits_{0}^{t} \frac{{f_{k}^{2} \left( z \right)}}{{f_{k}^{2} \left( 0 \right)}}e^{{\left( {\beta - 2\rho } \right)z}} dz.$$

Equation (22) has a closed-form expression in terms of elementary functions due to being the product of sine and cosine functions with an exponential function when $\beta \ne 2\rho$, or simply the product of sines and cosines when $\beta =2\rho$ (supplementary materials).

Furthermore, given (21) and (22) it follows that (14) converges to a periodic trajectory when $\beta -2\rho >0$ or the DFE when $\beta -2\rho \le 0$.

#### The ${\varvec{n}}$-Strain gSIS Model

We consider the same type of extension as in the $n$-strain SIS model. The transition of person-days of infection for an $n$-strain system is assumed to be governed by

23 $$\begin{gathered} \left( {\overline{m}S} \right)^{\prime} = N \overline{m}^{\prime} + \mathop \sum \limits_{j = 1}^{n} - \frac{{\beta_{j} }}{N}SI_{j} m_{j} + \frac{{m_{j}^{\prime} + 1}}{{m_{j} }}I_{j} m_{j} , \hfill \\ \left( {m_{j} I_{j} } \right)^{\prime} = \frac{{\beta_{j} }}{N}SI_{j} m_{j} - \frac{{m_{j}^{\prime} + 1}}{{m_{j} }}I_{j} m_{j} , \forall j \in \left\{ {1 \ldots n} \right\}, \hfill \\ \overline{m} = \mathop \sum \limits_{j = 1}^{n} m_{j} \frac{{I_{j} }}{{\mathop \sum \nolimits_{k = 1}^{n} I_{k} }}. \hfill \\ \end{gathered}$$

Provided each ${m}_{j}$ approaches a constant value sufficiently fast, the equilibria of system (23) can be found akin to that of the traditional $n$-strain SIS system, and consists of a DFE, single-strain equilibria, and co-existence equilibria.

### Stability Analysis

We now present the stability analysis of single-strain and $n$-strain SIS models, along with their gSIS counterparts when $m(t)$ is assumed to be of the form (20). Specifically, we show conditions when 1) the DFE is locally stable, 2) the single-strain and multi-strain endemic equilibria of the $n$-strain SIS model are locally stable when they exist, and 3) the stability of periodic solutions of the $n$-strain gSIS model.

#### The Single-Strain SIS Model

For simplicity, we eliminate ${S}^{\prime}$ in (1) due to the conservation of population. Starting with the DFE, assuming that ${\varepsilon }_{1}=I-\widehat{I}$, subbing in system (1), and neglecting higher order terms, we have that

24 $$\frac{d}{dt}\varepsilon_{1} \approx \left( {\beta - \gamma } \right)\varepsilon_{1} .$$

Thus, the DFE is locally stable provided $\beta -\gamma$ < 0.

Similarly, for ${\varepsilon }_{1}=I-\widetilde{I}$, we have that

25 $$\frac{d}{dt}\varepsilon_{1} \approx - \left( {\beta - \gamma } \right)\varepsilon_{1} ,$$

which is locally stable when $\beta -\gamma$ > 0.

Note, the stability of system (1) may also be inferred from other approaches, such as the next-generation method (Diekmann et al. 2010), which gives the threshold for the DFE to be stable as

26 $${\mathcal{R}}_{0} : = \frac{\beta }{\gamma } < 1.$$

Both equilibria's stability may also be directly inferred from the closed-form solution (2), as the sign of $\beta -\gamma$ controls whether the exponential term is either growth or decay.

#### The ${\varvec{n}}$-Strain SIS Model

For the $n$-strain SIS model, the stability of equilibria can be directly inferred from (8). Consider a subset $\Omega \subseteq \left\{1,\dots , n\right\}$ exists such that $\frac{{\beta }_{k}}{{\gamma }_{k}}=\underset{j}{\text{max}}\frac{{\beta }_{j}}{{\gamma }_{j}}$ for all $k\in\Omega$ and $\frac{{\beta }_{k}}{{\gamma }_{k}}>\frac{{\beta }_{l}}{{\gamma }_{l}}$ for $k\in\Omega$ and $\forall l\in \left\{1, \dots ,n\right\}\backslash\Omega .$ By (8), one can show all strains $l$ such that $\frac{{\beta }_{l}}{{\gamma }_{l}}<\frac{{\beta }_{k}}{{\gamma }_{k}}$ with $k\in\Omega$ tend to 0. In addition, (8) can also be used to decouple ${I}_{k}^{\prime}$ from all disease strains when the property that $\frac{{\beta }_{k}}{{\gamma }_{k}}=\frac{{\beta }_{j}}{{\gamma }_{j}}$ holds$.$ The resulting first-order nonlinear autonomous ODE is bounded (Martcheva 2009), so by the Poincaré-Bendixson theorem, it can only converge to an equilibrium.

**Fig. 2:**
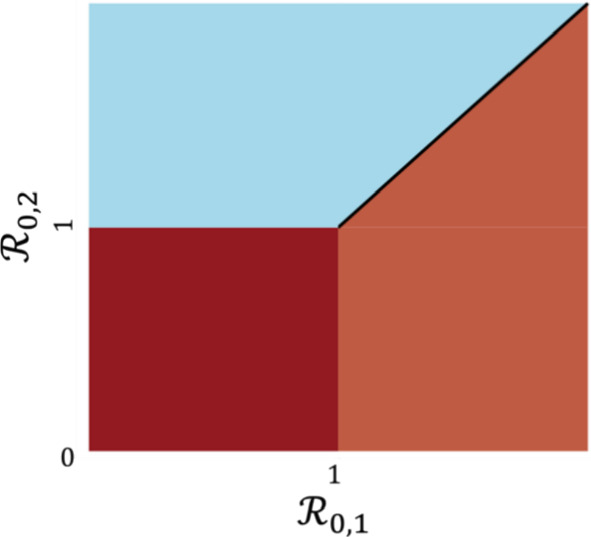
Stability regions for two-strain dynamics. The DFE is locally stable when ${\mathcal{R}}_{\text{0,1}}\le 1$ and ${\mathcal{R}}_{\text{0,2}}\le 1$ (brown region), the strain 1 dominant and strain 2 extinct solution is locally stable when ${\mathcal{R}}_{\text{0,1}}>1$ and ${\mathcal{R}}_{\text{0,2}}<{\mathcal{R}}_{0,1}$ (salmon region), the strain 2 dominant and strain 1 extinct solution is locally stable when ${\mathcal{R}}_{0,2}>1$ and ${\mathcal{R}}_{0,1}<{\mathcal{R}}_{0,2}$ (blue region), and the strain co-existence solution is locally stable when ${\mathcal{R}}_{0,j}=\frac{{\beta }_{j}}{2{\rho }_{j}}=\frac{{\beta }_{j}}{{\gamma }_{j}}>1$ (black line). For the two-strain SIS model, dominant and co-existence solutions are endemic equilibria, whereas they are periodic solutions in the two-strain gSIS model case (Color figure online)

It follows that ${I}_{j}\to 0$ if ${\beta }_{j}-{\gamma }_{j}\le 0$ and ${I}_{j}\to {\omega }_{j}N\left(1-\frac{{\gamma }_{j}}{{\beta }_{j}}\right)$ when ${\beta }_{j}-{\gamma }_{j}>0$ as $t\to \infty$, where $j\in\Omega$ and ${\omega }_{j}$ can be determined by (6) and (8). Naturally, for ${\beta }_{j}-{\gamma }_{j}>0, j\in\Omega ,$ a co-existence equilibrium occurs if $\left|\Omega \right|>1$, and a single-strain endemic and all other strains extinct equilibrium occurs otherwise (Fig. 2).

#### The Single-Strain gSIS Model

Assuming $m$ is periodic of the form (20), and $\beta -2\rho \ne 0$, then the gSIS has a DFE and a solution, which is potentially periodic, given by

27 $$\begin{gathered} I = \frac{{I_{0} \frac{{f_{k}^{2} \left( t \right)}}{{f_{k}^{2} \left( 0 \right)}}e^{{\left( {\beta - 2\rho } \right)t}} }}{{1 + \beta \frac{{I_{0} }}{N}\left( {\omega \left( t \right) - \omega \left( 0 \right)} \right)}}, \hfill \\ {\text{with}} \hfill \\ S = N - I, \hfill \\ \end{gathered}$$

where $\omega$(t) is a function composed of sines, cosines, and ${e}^{\left(\beta -2\rho \right)t}$, and defined in the supplementary materials for ease of presentation.

If $m$ is periodic of the form (20), and $\beta -2\rho =0$, then the gSIS has the solution

$$I = \frac{{I_{0} \frac{{f_{k}^{2} \left( t \right)}}{{f_{k}^{2} \left( 0 \right)}}}}{{1 + \beta \frac{{I_{0} }}{N}\left( {\xi \left( t \right) - \xi \left( 0 \right)} \right)}},$$

with

$$S = N - I,$$

where $\xi$(t) is a function composed of sines, cosines, and $\frac{1}{4}t$ (supplementary materials).

It follows that for the single-strain gSIS, the DFE is stable provided $\beta -2\rho \le 0$, and the periodic solution is stable when $\beta -2\rho >0$ (supplementary materials).

#### The ${\varvec{n}}$-Strain gSIS Model

For simplicity, using the conservation of population, we reduce system (27) to ${I}_{j}^{\prime}$ for $j\in \{1,\dots ,n\}$. Assuming that ${\varepsilon }_{j}={I}_{j}-{\widehat{I}}_{j}$, subbing in system (27), and neglecting higher order terms (Palmer 1975), we have that

28 $$\frac{d}{dt}\left( {\begin{array}{*{20}c} {\varepsilon_{1} } \\ \vdots \\ {\varepsilon_{n} } \\ \end{array} } \right) \approx A\left( t \right)\left( {\begin{array}{*{20}c} {\varepsilon_{1} } \\ \vdots \\ {\varepsilon_{n} } \\ \end{array} } \right),$$

where

$$\begin{gathered} A\left( t \right) = \hfill \\ \left( {\begin{array}{*{20}c} {\frac{{\beta_{1} }}{N}\left( {N - \widehat{I}_{1} - \mathop \sum \limits_{j = 1}^{n} \widehat{I}_{j} } \right) - \left( {\frac{{2m_{1} ^{\prime} + 1}}{{m_{1} }}} \right)} & { - \frac{{\beta_{1} }}{N}\widehat{I}_{1} } & \ldots & { - \frac{{\beta_{1} }}{N}\widehat{I}_{1} } \\ { - \frac{{\beta_{2} }}{N}\widehat{I}_{2} } & {\frac{{\beta_{2} }}{N}\left( {N - \widehat{I}_{2} - \mathop \sum \limits_{j = 1}^{n} \widehat{I}_{j} } \right) - \left( {\frac{{2m_{2} ^{\prime} + 1}}{{m_{2} }}} \right)} & \ldots & { - \frac{{\beta_{2} }}{N}\widehat{I}_{2} } \\ \vdots & \vdots & \ddots & \vdots \\ { - \frac{{\beta_{n} }}{N}\widehat{I}_{n} } & { - \frac{{\beta_{n} }}{N}\widehat{I}_{n} } & \ldots & {\frac{{\beta_{n} }}{N}\left( {N - \widehat{I}_{n} - \mathop \sum \limits_{j = 1}^{n} \widehat{I}_{j} } \right) - \left( {\frac{{2m_{n} ^{\prime} + 1}}{{m_{n} }}} \right)} \\ \end{array} } \right). \hfill \\ \end{gathered}$$

To determine when the DFE is locally stable we first define the diagonal matrix

29 $$\tilde{A}\left( t \right) = A\left( t \right)|_{{ I_{1} = 0, \ldots ,I_{n} = 0}} ,$$

and the matrix differential equation

30 $${\rm E}^{\prime } = \tilde{A}\left( t \right){\rm E},$$

with initial conditions $${\rm E}\left(0\right)={{\rm E}}_{0}=\left(\begin{array}{cc}1& 0\\ 0& 1\end{array}\right).$$

The monodromy matrix is then computed as

31 $$B = E_{0}^{ - 1} E\left( L \right) = E\left( L \right),$$

so that

32 $$E\left( {t + L} \right) = B \cdot E\left( t \right) { }\forall {\text{t}}.$$

As $\widetilde{A}\left(t\right)$ is a diagonal matrix, it follows (Tian and Wang 2015) that the characteristic multipliers are given by

33 $$\exp \left( {\mathop \smallint \limits_{0}^{t} \tilde{a}_{jj} dz} \right) = \exp \left( {\mathop \smallint \limits_{0}^{t} \beta_{j} - \frac{{2m_{j}^{\prime} + 1}}{{m_{j} }}dz} \right),$$

for $j\in \left\{1,\dots ,n\right\},$ and by (18), the Floquet exponents are

34 $$\lambda_{j} = \frac{1}{L}\mathop \smallint \limits_{0}^{L} \hat{a}_{jj} dz = \beta_{j} - 2\rho_{j} ,$$

for $$j\in \left\{1,\dots ,n\right\}$$.

From (43), we have that the DFE is locally stable when ${\lambda }_{j}={\beta }_{j}-2{\rho }_{j}<0 \forall j$.

To show that the DFE is also locally stable when ${\lambda }_{j}={\beta }_{j}-2{\rho }_{j}\le 0 \forall j,$ and ${\lambda }_{k}={\beta }_{k}-2{\rho }_{k}=0$ for at least one strain, we first note that

$$\mathop \smallint \limits_{0}^{L} \frac{d}{dt}\ln I_{k} dt = \mathop \smallint \limits_{0}^{L} \frac{{\beta_{k} }}{N}\left( {N - \mathop \sum \limits_{{j \in {\Omega } }} I_{j} } \right) - \left( {2\rho_{k} - 2\frac{{f_{n,k}^{\prime} \left( t \right)}}{{f_{n,k} \left( t \right)}}} \right)dt$$

and thus

$$I_{k} \left( L \right) = I_{k} \left( 0 \right)\exp \left( { - \frac{{\beta_{k} }}{N}\mathop \smallint \limits_{0}^{L} \left( {\mathop \sum \limits_{{j \in {\Omega } }} I_{j} } \right)dt} \right).$$

As $-\frac{{\beta }_{k}}{N}{\int }_{0}^{L}\left({\sum }_{j\in\Omega }{I}_{j}\right)dt<0$ when at least one ${I}_{j}>0,$ we have that ${I}_{k}\to 0$ as $t\to +\infty$.

To establish stability criteria for single- and multi-strain periodic solutions to (27), we first prove their existence and rule out the potential for chaotic behavior. Note that for any two strains,

35 $$\frac{{I_{j}^{\prime} }}{{\beta_{j} I_{j} }} - \frac{{I_{l}^{\prime} }}{{\beta_{l} I_{l} }} = - \frac{{2m_{j}^{\prime} + 1}}{{\beta_{j} m_{j} }} + \frac{{2m_{l}^{\prime} + 1}}{{\beta_{l} m_{l} }}.$$

Integrating (47) over $\left[0,t\right]$ yields,

36 $$\frac{1}{{\beta_{j} }}\left( {\ln \left( {I_{j} } \right) - \ln \left( {I_{j} \left( 0 \right)} \right)} \right) - \frac{1}{{\beta_{l} }}\left( {\ln \left( {I_{l} } \right) - \ln \left( {I_{l} \left( 0 \right)} \right)} \right) = - 2\left( {\frac{{\rho_{j} }}{{\beta_{j} }} - \frac{{\rho_{l} }}{{\beta_{l} }}} \right)t + \frac{2}{{\beta_{j} }}\ln \frac{{f_{k,j} \left( t \right)}}{{f_{k,j} \left( 0 \right)}} - \frac{2}{{\beta_{l} }}\ln \frac{{f_{k,l} \left( t \right)}}{{f_{k,l} \left( 0 \right)}}.$$

From (36), strains $l$ such that $\frac{{\beta }_{j}}{2{\rho }_{j}}>\frac{{\beta }_{l}}{2{\rho }_{l}}$ die out on account that ${I}_{j}<N$ is bounded, the right-hand side tends to $+\infty$, and consequently ${I}_{l}\to {0}^{+}.$ Further, if $\underset{j}{\text{max}}\frac{{\beta }_{j}}{2{\rho }_{j}}>\frac{{\beta }_{l}}{2{\rho }_{l}}$ for all $l\ne j$ then the system converges to the ${j}^{th}$ strain periodic and all other strains extinct solution, as described by (27) with ${I}_{l}=0, \forall l\ne j$. Alternatively, given a set $\Omega \subseteq \left\{1, \dots , n\right\}$ such that $\frac{{\beta }_{k}}{2{\rho }_{k}}=\underset{j}{\text{max}}\frac{{\beta }_{j}}{2{\rho }_{j}}$ for all $k\in\Omega$ and $\frac{{\beta }_{k}}{2{\rho }_{k}}>\frac{{\beta }_{l}}{2{\rho }_{l}}$ for $k\in\Omega , l\in \{1,\dots .,n\}\backslash\Omega$ then the right-hand side of (36) is periodic for strains in $\Omega$. Furthermore, each ${I}_{j}^{\prime}$ with $j\in\Omega$ can be decoupled from other strains through (36), along with imposing that the other disease strains have died out, namely ${I}_{l}=0$ for $l\in \{1,\dots .,n\}\backslash\Omega$. It follows that

37 $$I_{j}^{\prime} = \frac{{\beta_{j} }}{N}I_{j} \left( {N - I_{j} - \mathop \sum \limits_{{l \in {\Omega }\backslash j}} I_{l} \left( 0 \right)\frac{{f_{k,j}^{2} \left( t \right)}}{{f_{k,j}^{2} \left( 0 \right)}}\left( {\frac{{I_{j} }}{{I_{j} \left( 0 \right)}}} \right)^{{\frac{{\beta_{l} }}{{\beta_{j} }}}} \left( {\frac{{f_{k,l}^{2} \left( t \right)}}{{f_{k,l}^{2} \left( 0 \right)}}} \right)^{{\frac{{2\beta_{l} }}{{\beta_{j} }}}} } \right) - \left( {2\rho_{j} - \frac{{2f_{k,j}^{\prime} }}{{f_{k,j} }}} \right)I_{j} = :g\left( {t,I_{j} } \right),$$

or equivalently,

38 $$I_{j}^{\prime} = g\left( {z,I_{j} } \right), \frac{dz}{{dt}} = 1.$$

By the Poincaré-Bendixson theorem (38) cannot be chaotic, and thus (37) and (27) are not chaotic. Further, (23) is bounded (Martcheva 2009), so provided the DFE is unstable, namely ${\beta }_{j}-2{\rho }_{j}>0$ for at least one strain, it must converge to a periodic orbit.

As the $n$-strain gSIS model can be decoupled into $n$ first-order nonlinear non-autonomous ODEs, we can show the stability of its periodic solutions utilizing (37). Assuming strain-1 belongs to $\Omega$, linearizing (37) with ${\epsilon }_{1}={I}_{1}-{\widehat{I}}_{1}$, and neglecting higher-order terms of ${\epsilon }_{1}$, we have that

39 $$\begin{aligned}\epsilon_{1}^{\prime} = \,&\left( \beta_{1} - 2\rho_{1} - 2\frac{{\beta_{1} }}{N}\widehat{{I_{1} }} + \frac{{2f_{k,1}^{\prime} }}{{f_{k,1} }} - \mathop \sum \limits_{{j \in {\Omega } \backslash \left\{ 1 \right\} }} \left( {\frac{{\beta_{1} }}{N} + \frac{{\beta_{j} }}{N}} \right)I_{j} \left( 0 \right)\frac{{f_{k,1}^{2} \left( t \right)}}{{f_{k,1}^{2} \left( 0 \right)}}\right.\\ &\left.\left( {\frac{{f_{k,j}^{2} \left( 0 \right)}}{{f_{k,j}^{2} \left( t \right)}}} \right)^{{\frac{{2\beta_{j} }}{{\beta_{1} }}}} \left( {\frac{{\widehat{{I_{1} }}}}{{I_{1} \left( 0 \right)}}} \right)^{{\frac{{\beta_{j} }}{{\beta_{1} }}}} \right) \epsilon_{1} ,\end{aligned}$$

or equivalently, for the ease of presentation

40 $$\epsilon_{1}^{\prime} = a_{1} \left( t \right)\epsilon_{1} = \left( {\beta_{1} - 2\rho_{1} + \frac{{2f_{k,1}^{\prime} }}{{f_{k,1} }} - \mathop \sum \limits_{{j \in {\Omega } }} \left( {\frac{{\beta_{1} }}{N} + \frac{{\beta_{j} }}{N}} \right)\widehat{{I_{j} }}} \right) \epsilon_{1} .$$

The Floquet exponent is computed as

41 $$\lambda = \frac{1}{L}\mathop \smallint \limits_{0}^{L} a_{1} \left( t \right)dt = \beta_{1} - 2\rho_{1} - \frac{1}{L}\mathop \smallint \limits_{0}^{L} \mathop \sum \limits_{{j \in {\Omega } }} \left( {\frac{{\beta_{1} }}{N} + \frac{{\beta_{j} }}{N}} \right)\widehat{{I_{j} }} dz.$$

To simplify (41), first note that because ${I}_{1}\left(L\right)={I}_{1}\left(0\right)$, so we have

42 $$\mathop \smallint \limits_{0}^{L} \frac{d}{dt}\ln I_{1} dt = \mathop \smallint \limits_{0}^{L} \frac{{\beta_{1} }}{N}\left( {N - \mathop \sum \limits_{{j \in {\Omega } }} I_{j} } \right) - \left( {2\rho_{1} - 2\frac{{f_{k,1}^{\prime} \left( t \right)}}{{f_{k,1} \left( t \right)}}} \right)dt = 0,$$

and thus

43 $$\frac{1}{L}\mathop \smallint \limits_{0}^{L} \mathop \sum \limits_{{j \in {\Omega } }} I_{j} dt = N\left( {1 - \frac{{2\rho_{1} }}{{\beta_{1} }}} \right).$$

Furthermore, $\forall j\in\Omega$ there exists ${\omega }_{j}>0$ such that

44 $$\frac{1}{L}\mathop \smallint \limits_{0}^{L} I_{j} dt = \omega_{j} N\left( {1 - \frac{{2\rho_{k} }}{{\beta_{k} }}} \right),$$

where $${\sum }_{j\in\Omega }{\omega }_{j}=1$$.

Using (44), we have that (41) simplifies (supplementary materials) to

$$\lambda = \frac{1}{L}\mathop \smallint \limits_{0}^{L} a_{1} \left( t \right)dt = - \left( {\beta_{1} - 2\rho_{1} } \right)\frac{{\overline{\rho }}}{{\rho_{1} }},$$

with $$\overline{\rho }={\sum }_{k\in\Omega }{\rho }_{k}{\omega }_{k}$$.

Because $\frac{\overline{\rho }}{{\rho }_{1}}>0,$ strain 1 has a locally stable periodic orbit provided ${\beta }_{1}-2{\rho }_{1}>0$. It follows that a multi-strain periodic solution is stable provided ${\beta }_{j}-2{\rho }_{j}>0$ for all $j\in\Omega$ with ${\beta }_{l}-2{\rho }_{l}<0$ for $l\in \{1,\dots ,n\}\backslash\Omega$ (Fig. 2).

## Application of Methodology: Forecasting Syphilis in the US

To illustrate the behaviors of the gSIS model, we applied it to recent syphilis data from the US. We consider pre-COVID era syphilis incidence data from January 1st, 2018 to December 31st, 2019. We use the 2018 incidence data to calibrate parameters in the gSIS model, along with estimates of syphilis’ ${\mathcal{R}}_{0}\approx 1.5$ (Tsuzuki et al. 2018). We inform on the quality of model fit using Akaike information criterion (AIC), and utilize the 2019 incidence data to provide skill scores of model predictions for each modeling scenario. Further, as the US features a single dominant lineage, composing 81.3% of all syphilis cases (Beale et al. 2021), we consider modeling scenarios with a single-strain gSIS model with mean residual waiting-times based on a Fourier series with $k=$ 0 (i.e. the SIS model), 1, 2, 4, 8, 16, 32 and 64. All code used to illustrate findings is publicly available at (Greenhalgh 2022).

### Parameters Estimation and Model Evaluation

To estimate the values of $\gamma$ and $\beta$, we make use of estimates of the basic reproductive number, along with its next-generation method estimate. For the traditional SIS model, it follows that

$${\mathcal{R}}_{0} = \frac{\beta }{\gamma } = 1.5 \Leftrightarrow \beta = 1.5\gamma .$$

For the gSIS model, a similar calculation yields

$$\beta = 3\rho .$$

The population size is taken as $N=\text{15,500,000}$, which is approximately the sexually active population in the US (Farrell et al. 2023).

For the recovery term, we take $L=26$ weeks so that cosine and sine terms have a period of at least 52 weeks. In addition, we estimate parameters in the recovery term by minimizing the mean square error (MSE),

$${\text{MSE}}_{{{\text{ref}}}} \left( {\Theta } \right) = \frac{1}{52}\mathop \sum \limits_{t = 0}^{52} \left( {\lambda_{gSIS} \left( {t;{\Theta }} \right) - \lambda_{obs} \left( t \right)} \right)^{2} ,$$

where ${\lambda }_{obs}(t)$ is the observed new incidence in week $t$, ${\lambda }_{gSIS}\left(t;\Theta \right)=\frac{\beta }{N}I\left(N-I\right)$ is the estimated new incidence in week $t$ for parameter set ${\Theta }_{\text{k}}=\left({I}_{0},\rho ,{a}_{0},{a}_{1}, \dots , {a}_{k}, {b}_{1}, {b}_{2}, \dots ,{b}_{k}\right),$ where $k=0, 1, 2, 4, 8, 16, 32$ and $64$.

It follows that the optimal parameter sets for each scenario are given by

$${\hat{\Theta }}_{k} = \arg \mathop {\min }\limits_{{\Theta }} {\text{MSE}}_{{{\text{ref}}}} \left( {\Theta } \right)$$

$$s.t. \frac{{m^{\prime} + 1}}{m} \ge 0, f_{k} \left( t \right) > 0,$$

where the constraints ensure the mean residual waiting-time, hazard rates, and recovery term are biologically feasible and finite.

To assess the quality of each gSIS model fit to data relative to complexity, we utilize AIC. AIC values are determined as

$${\text{AIC}}_{{{\text{value}}}} \left( \Theta \right) = 2k + 52\left( {\log \left( {2\pi {\text{MSE}}_{{{\text{ref}}}} \left( \Theta \right)} \right) + 1} \right),$$

and AIC scores are calculated as

$$\Delta {\text{AIC}}_{j} = {\text{AIC}}_{{{\text{value}}}} \left( {\Theta_{j} } \right) - {\text{AIC}}_{{{\text{value}}}} \left( {\Theta_{0} } \right),$$

where ${\Theta }_{0}$ denotes the optimal parameter set for the traditional SIS model, and ${\Theta }_{j}$ denotes the optimal parameter set for the gSIS model when there are $j$ Fourier series terms.

In addition, we also inform on the predictive ability of each gSIS model using forecasting metrics (Murphy 1988).

Defining

$${\text{MSE}}_{{{\text{forecast}}}} \left( {{\Theta }_{j} ,n} \right) = \frac{1}{52}\mathop \sum \limits_{k = 53}^{104} \left( {\lambda_{gSIS} \left( {k;{\Theta }_{{\text{j}}} } \right) - \lambda_{obs} \left( k \right)} \right)^{2} ,$$

we estimate the quality of prediction by the Skill Scores (SS) as

$$SS\left( {{\hat{\Theta }}_{j} ,n} \right) = 1 - \frac{{{\text{MSE}}_{{{\text{forecast}}}} \left( {{\hat{\Theta }}_{j} ,n} \right)}}{{{\text{MSE}}_{{{\text{ref}}}} \left( {{\hat{\Theta }}_{j} } \right)}},$$

where $$n=\text{0,1},\text{2,4},\text{8,16}, 32,$$ or $$64.$$

To inform on the sensitivity of model predictions relative to parameters, we conducted a local sensitivity analysis of ${\mathcal{R}}_{0,j}$, and a global sensitivity analysis of the model fit relative to parameters.

For the local sensitivity analysis of ${\mathcal{R}}_{0,j}$, we calculated normalized forward sensitivity indices (Chitnis et al. 2008), namely

$$Y_{{\beta_{j} }}^{{{\mathcal{R}}_{0,j} }} = \frac{{\beta_{j} }}{{{\mathcal{R}}_{0,j} }} \cdot \frac{\partial }{{\partial \beta_{j} }}{\mathcal{R}}_{0,j} ,$$

and

$$Y_{{\gamma_{j} }}^{{{\mathcal{R}}_{0,j} }} = \frac{{\gamma_{j} }}{{{\mathcal{R}}_{0,j} }} \cdot \frac{\partial }{{\partial \gamma_{j} }}{\mathcal{R}}_{0,j} .$$

For the global sensitivity analysis, we calculated Sobol’ indices (Tosin et al. 2020). For this analysis, we consider the output as the log of the sum of square error of the gSIS model fit to 2018 syphilis incidence data in the US,

$$\log \left( {{\text{SSE}}\left( {\Theta } \right)} \right) = \log \left( {\sqrt {\mathop \sum \limits_{t = 0}^{52} \left( {\lambda_{gSIS} \left( {t;{\Theta }} \right) - \lambda_{obs} \left( t \right)} \right)^{2} } } \right),$$

with gSIS model parameters, $\Theta$, as the inputs (supplementary materials). Given this setup, we calculated the First-order indices, ${S}_{i}$, which indicate the contribution to output variance caused by uncertainty in the particular input parameter, and the Total-effect index, ${T}_{i}$, which measures the contribution to output variance caused by uncertainty in the input parameter and includes all variance caused by its interactions (Tosin et al. 2020) (supplementary materials).

### Simulations

#### Quality of Model Fit and Forecast Skill

**Fig. 3:**
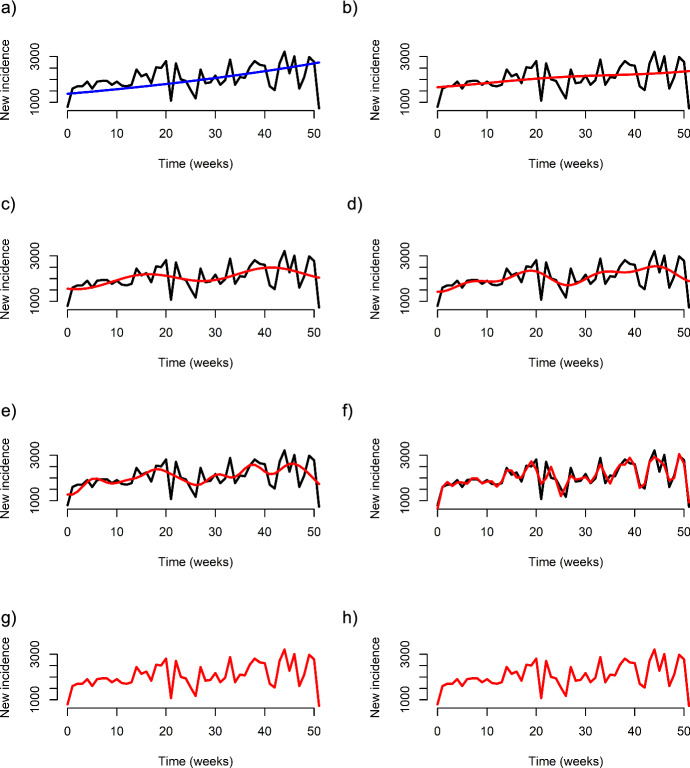
Best fits of SIS and gSIS models to 2018 syphilis incidence in the US. **a** The best fit of the SIS model (blue curve) to data, and **b**–**h** the best fit of the gSIS model for $k=1, 2, 4, 8, 16, 32,$ and $64$ (red curves), respectively, along with syphilis incidence data (black curve) (Color figure online)

Fitting each gSIS model scenario to the 2018 syphilis incidence data in the US resulted in $\text{MSE}$s that ranged from $3.07\times {10}^{5}$ to $1.68$ (Table 1), with the best-fit in the mean-square error sense corresponding to the $k=64$ scenario, although the $k=32$ scenario was the same due to rounding (Fig. 3). When accounting for model complexity through AIC, the lowest AIC score corresponded to the scenario when $k=32$ (Table 1).

**Table 1:** Quality of gSIS model fit to data and forecasting skill

Scenario	${\text{MS}}_{\text{ref}}$	${\text{MS}}_{\text{forecast}}$	$\text{AIC}$ score	Skill score
0	$3.07\times {10}^{5}$	$3311010.8$	661.1	$-9.77$
1	$2.54\times {10}^{5}$	$687809.8$	657.2	$-1.70$
2	$2.26\times {10}^{5}$	$408307.2$	655.0	$-0.81$
4	$2.10\times {10}^{5}$	$371543.8$	659.4	$-0.77$
8	$1.93\times {10}^{5}$	$320889.0$	670.9	$-0.66$
16	$6.73\times {10}^{4}$	$329296.6$	648.0	$-3.89$
32	$1.68$	$411894.4$	161.9	$-2.45\times {10}^{5}$
64	$1.68$	$411894.4$	289.1	$-2.45\times {10}^{5}$

**Fig. 4:**
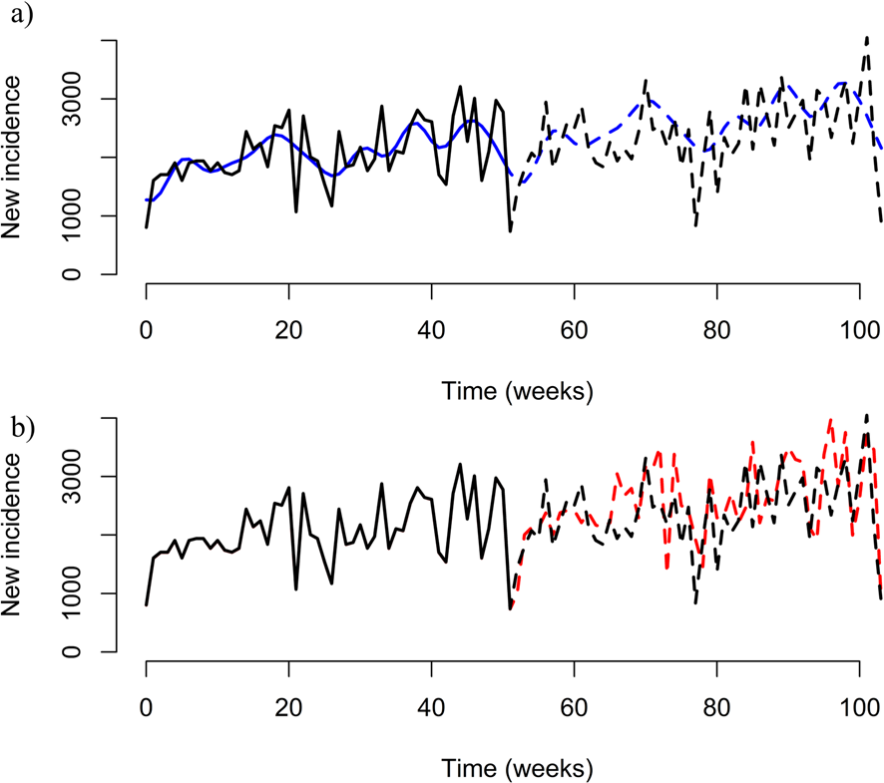
Fitted and predicted trajectory of syphilis. Trajectories of the gSIS model with **a** the lowest skill score, $k=8$ (blue solid and dashed curves), and **b** the lowest AIC score, $k=32$ (red solid and dashed curves), with 2018 syphilis incidence data in the US (black solid curve) and 2019 syphilis incidence data in the US (black dashed curve) (Color figure online)

With regard to predicting future trends of syphilis, skill scores ranged from a minimum of $-2.45\times {10}^{5}$ to a maximum of $-0.66$ (Table 1). The optimal skill score occurred when $k=8$ (Fig. 4), with the associated parameter values provided in Table 2. The scenarios where $k\ge 32$ had the lowest skill scores of the scenarios considered.

**Table 2:** Parameters for the SIS and gSIS-8 model

Parameter	Symbol	Base value(s)
*Transmission rate*		
SIS model	$\beta$	${\mathcal{R}}_{0}\gamma {\text{ day}}^{-1}$
gSIS-8 model	$\beta$	${\mathcal{R}}_{0}\left(2\rho \right) {\text{day}}^{-1}$
Avg. duration of infection	$1/\gamma$	$254.4 \text{ days}$
Avg. duration of infection in the absence of periodic effects	$1/(2\rho )$	$111.9 \text{ days}$
Even amplitudes of periodic effects	${a}_{0}$	$8.70\times {10}^{-1}$
	${a}_{1}$	$-3.86\times {10}^{-4}$
	${a}_{2}$	$-5.34\times {10}^{-4}$
	${a}_{3}$	$6.14\times {10}^{-5}$
	${a}_{4}$	$1.43\times {10}^{-5}$
	${a}_{5}$	$-9.15\times {10}^{-5}$
	${a}_{6}$	$6.60\times {10}^{-5}$
	${a}_{7}$	$7.93\times {10}^{-6}$
	${a}_{8}$	$-6.64\times {10}^{-5}$
Odd amplitudes of periodic effects	${b}_{1}$	$5.26\times {10}^{-4}$
	${b}_{2}$	$8.33\times {10}^{-4}$
	${b}_{3}$	$-4.78\times {10}^{-5}$
	${b}_{4}$	$3.50\times {10}^{-4}$
	${b}_{5}$	$1.70\times {10}^{-4}$
	${b}_{6}$	$1.32\times {10}^{-4}$
	${b}_{7}$	$-3.29\times {10}^{-5}$
	${b}_{8}$	$3.01\times {10}^{-6}$
Period of seasonality	$2L$	$52$ weeks
Sexually active population in US	$N$	$15.5 \text{ million people}$
Basic reproductive number	${\mathcal{R}}_{0}$	$1.5$

#### Sensitivity analysis

Due to the formulation of ${\mathcal{R}}_{0,j}$, its normalized forward sensitivity indices are ${Y}_{{\beta }_{j}}^{{\mathcal{R}}_{0,j}}=1,$ and ${Y}_{{\gamma }_{j}}^{{\mathcal{R}}_{0,j}}=-1.$ ${\mathcal{R}}_{0,j}$. This indicates that an arbitrary percent increase in ${\beta }_{j}$ will result in the same arbitrary percent increase for ${\mathcal{R}}_{0,j}$. Alternatively, an arbitrary percent increase in ${\gamma }_{j}$ will have the opposite effect, and result in the same arbitrary percent decrease for ${\mathcal{R}}_{0,j}$.

**Fig. 5:**
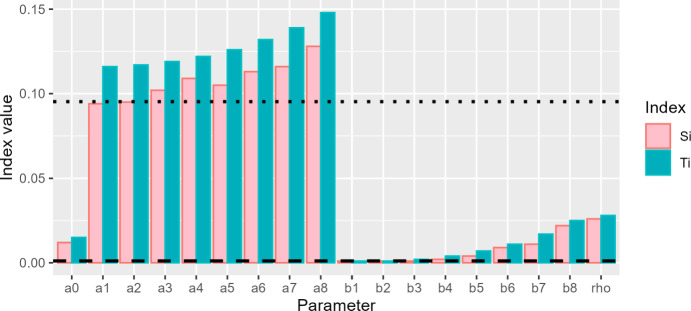
Sobol’ First-order and Total-effect indices. The First-order index, ${S}_{i}$, and Total-effect index, ${T}_{i}$ for the model parameters, along with the First-order index (black dashed line) and the Total-effect index (black dotted line) for a dummy variable that does not affect model output (Color figure online)

For the Sobol’ indices, our results indicate that uncertainty in the even amplitudes of periodic effects, ${a}_{k}$ with $k\ge 1$, had the greatest effect on sum of square error, as indicated by their First-order indices and Total-effect indices (Fig. 5). Of these indices the parameter attributed to the sine term with the smallest frequency, namely ${a}_{8}$, was most influential (Fig. 5). At the other end of the spectrum, the odd amplitudes of periodic effects, ${b}_{k}$ with $k\ge 1$ had minimal effect. In particular, the effects of ${b}_{1}, {b}_{2}$, and ${b}_{3}$ were less than the variation in the sum of square error caused by a dummy parameter, indicating these parameters are not significant (Fig. 5).

With regard to the capacity of First-order indices to explain the variation in the sum of square error, we have that ${\sum }_{i\in {\Theta }_{8}}{S}_{i}=0.93<1$ (Fig. 5), which implies that the model is non-additive (Puy et al. 2022) and that First-order indices are responsible for 93% of the variation in the sum of square error. Thus, higher order effects, which account for the interactions between parameters, are responsible only for about 7% of the variation.

## Discussion

We developed and analyzed an $n$-strain gSIS model that describes the evolution of the quantity, person-days of infection, illustrating its possible solution behaviors with a flexible mean residual waiting-time. Useful aspects of our work include the use of a mean residual waiting-time that led to a single-strain gSIS model with a closed-form solution, Floquet exponents that are algebraic expressions, which inform on the stability of the strain $j$ dominant and all other strains extinct periodic solutions, in addition to the stability of multi-strain coexistence periodic solutions. Finally, we applied our gSIS model to describe the trajectory of syphilis in the US, providing insights on periods important in its transmission cycle with respect to the quality of model fit to data, and with respect to forecasting future disease trends.

While we imposed a particular formulation of the mean residual waiting-time, and thus recovery rate, its overall form can describe many periodic recovery rates. To elaborate, if a general periodic recovery rate was desired, say $h\left(t\right)=h(t+L)$, then the Fourier series terms in $\left(2{m}^{\prime}+1\right)/m=2\rho -2{f}_{n}^{\prime}/{f}_{n}$ could approximate $h\left(t\right)$ through $\frac{{f}_{n}\left(t\right)}{{f}_{n}\left(0\right)}\sim \text{exp}\left(2\rho t+{\int }_{0}^{t}h\left(z\right)dz\right).$ This generality implies our $m(t)$ can approximate many duration of infection distributions. Thus, by applying a similar modification to the transmission probability, it is feasible that one could develop a non-autonomous gSIS model that closely mimics the dynamics of more complicated compartmental model. For example, through such modifications, one could set up an interval of the infectious period distribution that aligns with no transmissibility, which would essentially be equivalent to a removed compartment. An advantage of this approach is the resulting reduction in the dimension of the compartmental model, along with the potential for it to have a closed-form solution in terms of elementary functions. Thereby, our work may provide a simpler approach to investigating infectious disease dynamics, at least with respect to the order of the differential equation system.

With modest modifications our gSIS model could be applied to describe pathogens with similar transmission dynamics to syphilis, including other STIs, such as gonorrhea and chlamydia. For more complex transmission dynamics the gSIS model may not be applicable, although the idea behind the development of the gSIS model naturally extends to include additional compartments, with recent examples including a gSIR model that describes measles transmission in Iceland (Greenhalgh and Rozins 2021), and a gSEAIR model of chlamydia infection in the US (Farrell et al. 2023).Unsurprisingly, our work indicates that models with the highest quality of fit to data, whether that is measured by MSE or AIC, may be poor predictors of future trends due to overfitting, as illustrated with skill score. Traditionally, the use of skill score is reserved for predicted trends by stochastic models. Here, we illustrate that they may also have utility in informing on deterministic models’ predictions, particularly in the realm of infectious disease dynamics.

An interesting finding from our sensitivity analysis is that the even amplitudes of periodic effects were more influential on the variation in sum of square error then their odd counterparts, despite the average amplitude of the odd amplitudes of periodic effects being approximately twice that of the even ones (supplementary materials). While there may be many reasons for this, a potential explanation lays in the formulation of the integrating factor for the single-strain gSIS model, as it’s normalized by only the even Fourier coefficients (supplementary materials). On the other hand, of the influential First-order indices, it was unsurprising that ${a}_{8}$ had the greatest impact as it corresponded to the Fourier series term with the smallest period of 6.5 weeks. In the development and analysis of our gSIS model, we utilize several assumptions. First and foremost, the SIS models imply no immunity, but the literature suggests repeated infections with syphilis may confer limited immunity (Marchese et al. 2022). In addition, we do not account for individuals unaware of their infection, leading to underpredicting and inaccurate representation of syphilis’ trajectory. Another simplifying assumption is the lack of incorporation of specific age and gender demographics, as these are known factors in STI transmission (Tao et al. 2020). Furthermore, the influence of social factors, such as the impact of COVID-19 on sexual behavior and healthcare access was not built into the model. Nonetheless, these weaknesses emphasize the opportunity to modify our model’s transmission rate or its compartmental structure in order to provide greater insight into STI transmission.

In summary, we developed a versatile framework capable of modeling a broad spectrum of disease trajectories through a closed-form periodic solution. The simplicity of our framework enables the exact computation of Floquet exponents, facilitating the stability analysis of multi-strain periodic solutions—a feature rarely achieved in compartmental models with time-varying rates without relying on numerical methods. By combining accessibility with analytical rigor, our framework thus potentially empowers the disease-modeling community by providing a practical and effective tool for exploring recurrent disease outbreaks.

## Supplementary Information

Below is the link to the electronic supplementary material.

Supplementary file1 (DOCX 1259 KB)

## Data Availability

The datasets generated and/or analyzed during the current study are available in a GITHUB repository (see 10.5281/zenodo.6595905).
