# Supplementary material for: Dynamics and Persistence of a Generalized Multi-strain SIS Model
Source: Bull Math Biol. 2025 Sep 10;87(10):147. doi: 10.1007/s11538-025-01516-z (PMC12423140; doi:10.1007/s11538-025-01516-z)
Supplement: Supplementary file 1 — Supplementary file1 (DOCX 1259 KB) [file 11538_2025_1516_MOESM1_ESM.docx]

**Supplementary Materials**

Dynamics and persistence of a generalized multi-strain SIS model

Scott Greenhalgh, Tabitha Henriquez, Michael Frutschy, Rebecah Leonard

This appendix provides detailed information supporting our generalized susceptible-infected-susceptible (gSIS) model analysis. Specifically, we provide details about the closed-form periodic solution of our gSIS model when $\beta-2\rho\neq0$, and when $\beta-2\rho\neq0$, along with details on how we obtained algebraic expressions for the Floquet exponents, and the infectious period distribution, duration of infection distribution, and hazard rate. We also provide further details pertaining to the calculation of Sobol’ indices.

The solution to the single-strain gSIS model (13) is

$$I\left( t \right)=\frac{I_{0}\mu\left( t \right)}{1+\frac{\beta}{N}I_{0\int_{0}^{t} \mu\left( z \right)dz}},$$

where the general form of the integrating factor is

$$\mu\left( t \right) =\frac{\left( \frac{a_{0}}{2}+\sum_{k=1}^{n} a_{k}\cos\left( \frac{k\pi t}{L} \right)+b_{k}\sin\left( \frac{k\pi t}{L} \right) \right)^{2}}{\left( \frac{a_{0}}{2}+\sum_{k=1}^{n} a_{k} \right)^{2}}e^{\left( \beta-2\rho\right)t}.$$

**S1. The case when** $\boldsymbol{\beta-2}\boldsymbol{\rho\neq0.}$ Given $\beta-2\rho\neq0$, it follows that

$$\int_{0}^{t} \mu\left( z \right)dz=\frac{1}{\left( \frac{a_{0}}{2}+\sum_{k=1}^{n} a_{k} \right)^{2}}\int_{0}^{t} \left( \frac{a_{0}}{2}+\sum_{k=1}^{n} a_{k}\cos\left( \frac{k\pi z}{L} \right)+b_{k}\sin\left( \frac{\pi kz}{L} \right) \right)^{2}e^{\left( \beta-2\rho\right)z}dz.$$

We thus have that

$$\int_{0}^{t} \mu\left( z \right)dz=\omega\left( t \right)-\omega\left( 0 \right)=\frac{1}{\left( \frac{a_{0}}{2}+\sum_{k=1}^{n} a_{k} \right)^{2}}\left( T_{1}a_{0}^{2}+T_{2}a_{0}+T_{3} \right),$$

where the coefficient of $a_{0}^{2}$ is

$$T_{1}=\int_{0}^{t} \frac{1}{4}e^{\left( \beta-2\rho\right)z}dz=\frac{1-e^{\left( \beta-2\rho\right)t}}{4\left( 2\rho-\beta\right)},$$

and the coefficient of $a_{0}$ is

$$T_{2}=\sum_{k=1}^{n} W_{0,k}+W_{1,k}\cos\left( \frac{k\pi t}{L} \right)+W_{2,k}\sin\left( \frac{k\pi t}{L} \right),$$

where

$$W_{0,k}=\frac{\left( a_{k}\left( \beta-2\rho\right)L+b_{k}k\pi\right)}{\left( \beta-2\rho\right)^{2}L^{2}+k^{2}\pi^{2}}L,$$

$$W_{1,k}=\frac{\left( a_{k}\left( \beta-2\rho\right)L+b_{k}k\pi\right)}{\left( \beta-2\rho\right)^{2}L^{2}+k^{2}\pi^{2}}e^{\left( \beta-2\rho\right)t}L,$$

and

$$W_{2,k}=\frac{\left( b_{k}\left( \beta-2\rho\right)L+a_{k}k\pi\right)}{\left( \beta-2\rho\right)^{2}L^{2}+k^{2}\pi^{2}}e^{\left( \beta-2\rho\right)t}L.$$

The term $T_{3}$ is given by

$$T_{3}=\sum_{j=1}^{n} \sum_{k=1}^{n} V_{0,j,k}+V_{1,j,k}\cos\left( \frac{(j-k)\pi t}{L} \right)+V_{2,j,k}\cos\left( \frac{\left( j+k \right)\pi t}{L} \right)+V_{3,j,k}\sin\left( \frac{(j-k)\pi t}{L} \right)+V_{4,j,k}\sin\left( \frac{\left( j+k \right)\pi t}{L} \right),$$

where

$$V_{0,j,k}=-\frac{1}{2}\frac{L\left( \left( \beta-2\rho\right)\left( a_{k}a_{j}+b_{j}b_{k} \right)L+\pi\left( a_{j}b_{k}-a_{k}b_{j} \right)\left( j-k \right) \right)}{\left( \beta-2\rho\right)^{2}L^{2}+\left( j+k \right)^{2}\pi^{2}}-\frac{1}{2}\frac{L\left( \left( \beta-2\rho\right)\left( a_{k}a_{j}-b_{j}b_{k} \right)L-\pi\left( a_{j}b_{k}+a_{k}b_{j} \right)\left( j+k \right) \right)}{\left( \beta-2\rho\right)^{2}L^{2}+\left( j+k \right)^{2}\pi^{2}},$$

$$V_{1,j,k}=\frac{1}{2}\frac{L\left( \left( \beta-2\rho\right)\left( a_{k}a_{j}+b_{j}b_{k} \right)L+\pi\left( a_{j}b_{k}-a_{k}b_{j} \right)\left( j-k \right) \right)}{\left( \beta-2\rho\right)^{2}L^{2}+\left( j+k \right)^{2}\pi^{2}}e^{\left( \beta-2\rho\right)t},$$

$$V_{2,j,k}=\frac{1}{2}\frac{L\left( \left( \beta-2\rho\right)\left( a_{k}a_{j}-b_{j}b_{k} \right)L-\pi\left( a_{j}b_{k}+a_{k}b_{j} \right)\left( j+k \right) \right)}{\left( \beta-2\rho\right)^{2}L^{2}+\left( j+k \right)^{2}\pi^{2}}e^{\left( \beta-2\rho\right)t},$$

$$V_{3,j,k}=\frac{1}{2}\frac{L\left( \left( \beta-2\rho\right)\left( a_{k}b_{j}-a_{j}b_{k} \right)L+\pi\left( a_{k}a_{j}+b_{k}b_{j} \right)\left( j-k \right) \right)}{\left( \beta-2\rho\right)^{2}L^{2}+\left( j-k \right)^{2}\pi^{2}}e^{\left( \beta-2\rho\right)t},$$

and

$$V_{4,j,k}=\frac{1}{2}\frac{L\left( \left( \beta-2\rho\right)\left( a_{k}b_{j}+a_{j}b_{k} \right)L+\pi\left( a_{k}a_{j}-b_{k}b_{j} \right)\left( j+k \right) \right)}{\left( \beta-2\rho\right)^{2}L^{2}+\left( j+k \right)^{2}\pi^{2}}e^{\left( \beta-2\rho\right)t}.$$

**S2. The case when** $\boldsymbol{\beta-2}\boldsymbol{\rho=0.}$ Under the assumption $\beta-2\rho=0$, the integrating factor becomes

$$\mu\left( t \right) =\frac{\left( \frac{a_{0}}{2}+\sum_{k=1}^{n} a_{k}\cos\left( \frac{k\pi t}{L} \right)+b_{k}\sin\left( \frac{k\pi t}{L} \right) \right)^{2}}{\left( \frac{a_{0}}{2}+\sum_{k=1}^{n} a_{k} \right)^{2}}.$$

It follows that

$$\int_{0}^{t} \mu\left( z \right)dz=\frac{1}{\left( \frac{a_{0}}{2}+\sum_{k=1}^{n} a_{k} \right)^{2}}\int_{0}^{t} \left( \frac{a_{0}}{2}+\sum_{k=1}^{n} a_{k}\cos\left( \frac{k\pi z}{L} \right)+b_{k}\sin\left( \frac{\pi kz}{L} \right) \right)^{2}dz$$

Evaluating the integral yields

$$\int_{0}^{t} \mu\left( z \right)dz=\xi\left( t \right)-\xi\left( 0 \right)=\frac{1}{\left( \frac{a_{0}}{2}+\sum_{k=1}^{n} a_{k} \right)^{2}}\left( H_{1}a_{0}^{2}+H_{2}a_{0}+H_{3} \right)$$

where the coefficient of $a_{0}^{2}$ is

$$H_{1}=\frac{1}{4}t,$$

the coefficient of $a_{0}$ is

$$H_{2}=\sum_{k=1}^{n} \frac{L}{k\pi}\left( a_{k}\sin\left( \frac{k\pi t}{L} \right)-b_{k}\left( \cos\left( \frac{k\pi t}{L} \right)-1 \right) \right),$$

and the term $H_{3}$ is given by

$$H_{3}=\sum_{j=1}^{n} \sum_{k=1}^{n} G_{0,j,k}+G_{1,j,k}\cos\left( \frac{(j-k)\pi t}{L} \right)+G_{2,j,k}\cos\left( \frac{\left( j+k \right)\pi t}{L} \right)+G_{3,j,k}\sin\left( \frac{(j-k)\pi t}{L} \right)+G_{4,j,k}\sin\left( \frac{\left( j+k \right)\pi t}{L} \right),$$

where

$$G_{0,j,k}=\frac{\left( j a_{k}b_{j}-kb_{k}a_{j} \right)L}{\pi\left( j-k \right)\left( j+k \right)},$$

$$G_{1,j,k}=\frac{1}{2}\frac{\left( b_{k}a_{j}-a_{k}b_{j} \right)L}{\pi\left( j-k \right)},$$

$$G_{2,j,k}=-\frac{1}{2}\frac{\left( a_{k}b_{j}-b_{k}a_{j} \right)L}{\pi\left( j+k \right)},$$

$$G_{3,j,k}=\frac{1}{2}\frac{\left( a_{k}a_{j}-b_{k}b_{j} \right)L}{\pi\left( j-k \right)},$$

and

$$G_{4,j,k}=\frac{1}{2}\frac{\left( a_{k}a_{j}-b_{k}b_{j} \right)L}{\pi\left( j+k \right)}.$$

**S3. The Floquet exponent for strain co-existence.** Here, we provide further details on the calculation of the Floquet exponent as characterized by (41), namely

$$\lambda_{1}=\frac{1}{L}\int_{0}^{L} a_{1}\left( t \right)dt=\beta_{1}-2\rho_{1}-\frac{1}{L}\int_{0}^{L} 2\frac{\beta_{1}}{N}\hat{I_{1}}+\sum_{j\in\Omega\backslash\{1\}} \left( \frac{\beta_{1}}{N}+\frac{\beta_{j}}{N} \right)\hat{I_{j}} dz,$$

which simplifies to

$$\lambda_{1}=\frac{1}{L}\int_{0}^{L} a_{1}\left( t \right)dt=\beta_{1}-2\rho_{1}-\frac{1}{L}\int_{0}^{L} \sum_{j\in\Omega} \left( \frac{\beta_{1}}{N}+\frac{\beta_{j}}{N} \right)\hat{I_{j}} dz.$$

To further simplify (41), we make use of (43),

$$\frac{1}{L}\int_{0}^{L} \sum_{j\in\Omega} I_{j}\left( z \right)dz=N\left( 1-\frac{2\rho_{k}}{\beta_{k}} \right), for any k\in\Omega,$$

and (44),

$$\frac{1}{L}\int_{0}^{L} I_{j}\left( z \right)dz=\omega_{j}N\left( 1-\frac{2\rho_{k}}{\beta_{k}} \right),$$

where $k\in\Omega,$ $\sum_{j\in\Omega} \omega_{j}=1$ and $\omega_{j}>0$ $\forall j\in\Omega$.

Further defining $\mathcal{R}_{0}=\frac{\beta_{k}}{2\rho_{k}}$, it follows upon multiplication by $2\rho_{k}\omega_{k}$ and then summation over $k\in\Omega$ that

| $\sum_{k\in\Omega} 2\mathcal{R}_{0}\rho_{k}\omega_{k}=\sum_{k\in\Omega} \beta_{k}\omega_{k}\Rightarrow\mathcal{R}_{0}=\frac{\sum_{k\in\Omega} \beta_{k}\omega_{k}}{\sum_{k\in\Omega} 2\rho_{k}\omega_{k}}=\frac{\bar{\beta}}{2\bar{\rho}}.$ | (S1) |
| --- | --- |

where $\sum_{k\in\Omega} \beta_{k}\omega_{k}=\bar{\beta},$ and $\sum_{k\in\Omega} \rho_{k}\omega_{k}=\bar{\rho}.$

Substituting (43) and (44) in $\lambda_{1}$, we have that

$$\lambda_{1}=\beta_{1}-2\rho_{1}-\sum_{j\in\Omega} \left( \frac{\beta_{1}}{N}+\frac{\beta_{j}}{N} \right)\omega_{j}N\left( 1-\frac{2\rho_{l}}{\beta_{l}} \right).$$

Noting that

$$\sum_{j\in\Omega} \frac{\beta_{1}}{N}\omega_{j}N\left( 1-\frac{2\rho_{l}}{\beta_{l}} \right)=\beta_{1}-2\rho_{1},$$

implies

$$\lambda_{1}=-\sum_{j\in\Omega} \beta_{j}\omega_{j}\left( 1-\frac{2\rho_{l}}{\beta_{l}} \right).$$

Factoring out $1-\frac{2\rho_{l}}{\beta_{l}}$ and using (S1) yields

$$\lambda_{1}=-\left( 1-\frac{2\bar{\rho}}{\bar{\beta}} \right)\bar{\beta}.$$

After distributing and simplifying the sum, it follows that

$$\lambda_{1}=-\left( \bar{\beta}-2\bar{\rho} \right).$$

Using $\bar{\beta}=2\bar{\rho}\mathcal{R}_{0}$, factoring, and then using $\mathcal{R}_{0}=\frac{\beta_{1}}{2\rho_{1}}$ yields,

$$\lambda_{1}=-\left( \mathcal{R}_{0}-1 \right)2\bar{\rho}=-\left( \beta_{1}-2\rho_{1} \right)\frac{\bar{\rho}}{\rho_{1}}.$$

As $\bar{\rho}/\rho_{1}>0,$ it follows that

$$\lambda_{1}<0 \mathrm{iff} \beta_{1}-2\rho_{1}>0.$$

**S4. Distributions and hazard rate.** Given the mean residual waiting-time (20), the infectious period distribution is defined as

$$Q\left( t \right)=\frac{m\left( 0 \right)}{m\left( t \right)}\exp\left( -\int_{0}^{t} \frac{1}{m\left( z \right)}dz \right)=\frac{m\left( t \right)}{m\left( 0 \right)}\frac{f_{k}\left( t \right)^{2}}{f_{k}\left( 0 \right)^{2}}e^{-2\rho t},$$

and the duration of infection distribution is

$$P\left( t,x \right)=\frac{m\left( x \right)}{m\left( t \right)}\exp\left( -\int_{x}^{t} \frac{1}{m\left( z \right)}dz \right)=\frac{m\left( t \right)}{m(x)}\frac{f_{k}\left( t \right)^{2}}{f_{k}\left( x \right)^{2}}e^{-2\rho t}.$$

In addition, provided the definition of the hazard rate,

$$\eta=\frac{m^{'}+1}{m},$$

we have that

$$\eta=\rho-\frac{f_{k}^{'}\left( t \right)}{f_{k}(t)}+\frac{f_{k}\left( t \right)e^{-\rho t}}{2m\left( 0 \right)f_{k}\left( 0 \right)+\int_{0}^{t} f_{k}(z)e^{-\rho z}dz}.$$

**S5. Calculation of Sobel’ indices.** Here we detail the sensitivity of the gSIS-8 model’s fit to the 2018 incidence data, using the log of the sum of square error (SSE) as

| $\log\left( \mathrm{SSE}\left( \Theta_{8} \right) \right)=\log\left( \sqrt{\sum_{t=0}^{52} \left( \lambda_{gSIS}\left( t;\Theta_{8} \right)-\lambda_{obs}\left( t \right) \right)^{2}} \right),$ | (S2) |
| --- | --- |

where $\lambda_{obs}\left( t \right)$ is the observed new incidence in week $t$, $\lambda_{gSIS}\left( t;\Theta_{8} \right)=\frac{\beta}{N}I\left( N-I \right)$ is the estimated new incidence in week $t$ as determined by the gSIS-8 model (11), where the parameter set $\Theta_{8}=\left( I_{0},\rho,a_{0},a_{1}, \ldots, a_{8}, b_{1}, b_{2}, \ldots,b_{8} \right)$ follows the distributions outlined in Table S1.

We inform on the model’s sensitivity to parameters through the calculation of Sobol incidences (Tosin et al. 2020) using the package sensobol (Puy et al. 2022) in R. For this calculation, we assume sample sizes of $K=300000$. We also assume the rate of recovery in the absence of periodic exponentially distributed (Table S1), and base the variance of the amplitude parameters on their collective sample variance (Table S1), namely

$$\sigma_{ab}^{2}=\frac{1}{16}\left( \left( a_{0}+\bar{x}_{ab} \right)^{2}+\sum_{j=1}^{8} \left( a_{j}+\bar{x}_{ab} \right)^{2}+\left( b_{j}+\bar{x}_{ab} \right)^{2} \right),$$

where $\bar{x}_{ab}=\frac{1}{17}\left( a_{0}+\sum_{j=1}^{8} a_{j}+b_{j} \right).$

Further details of parameter distributions are available in Table S1.

For our calculation, we have that the output, $\log\left( \mathrm{SSE}\left( \Theta_{8} \right) \right)$, is a unimodal distribution with an average of 13.35, and sample variance of 0.54 (Figure S1). In addition, when examining the variation of model output relative to parameters, there does not appear to be bifurcation points (Figure S2).


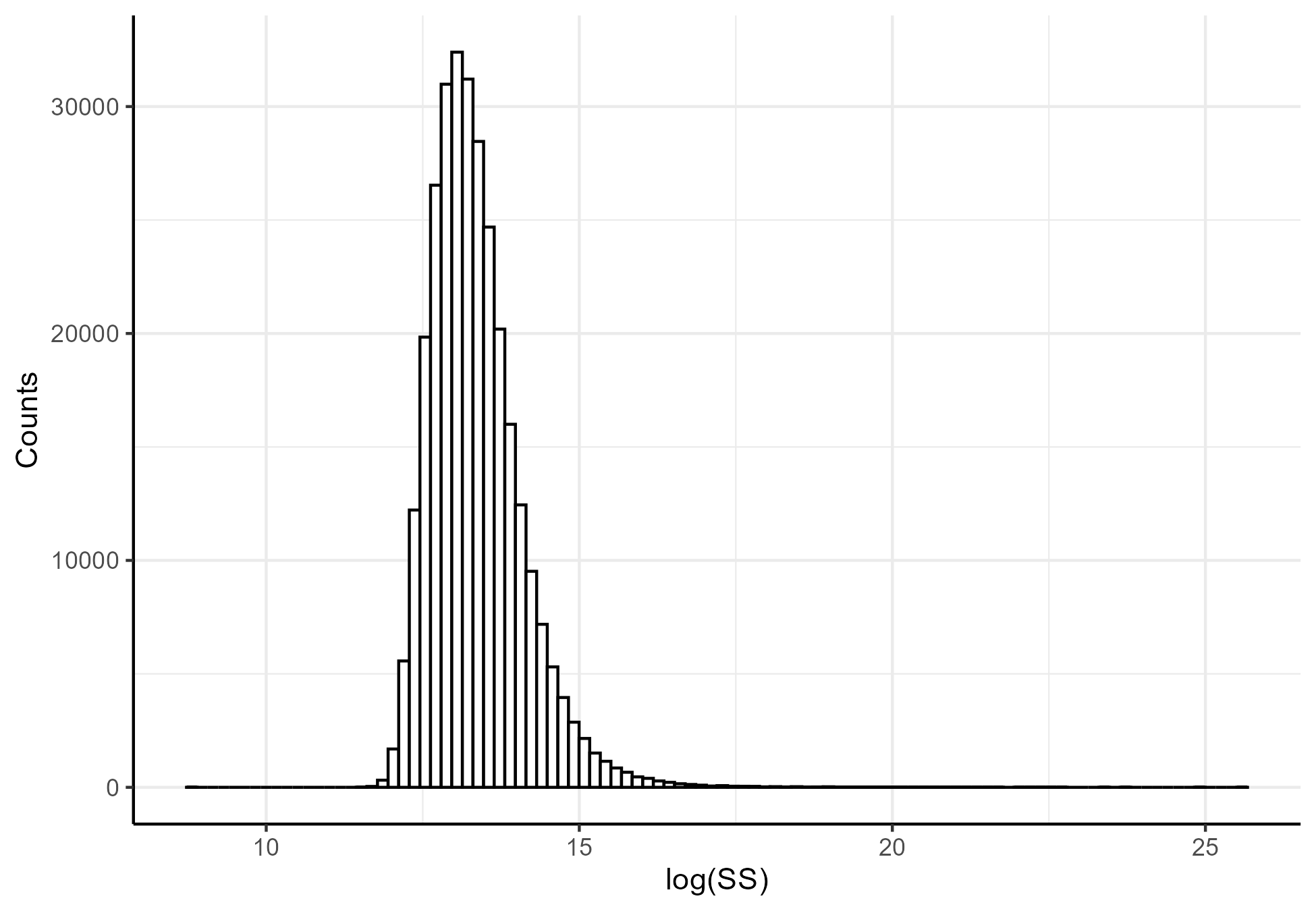


Figure S1. Empirical distribution of equation S2 output.


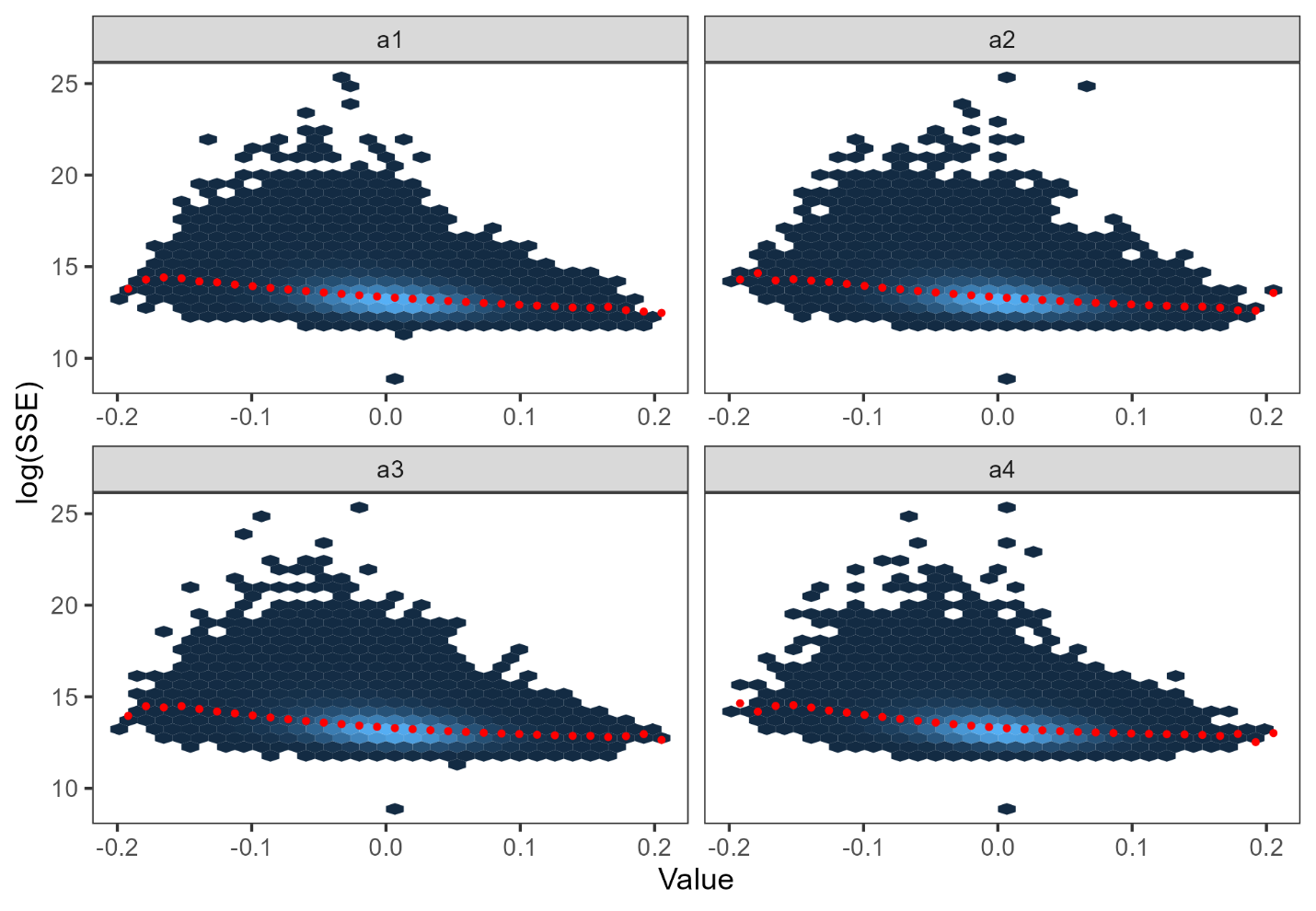

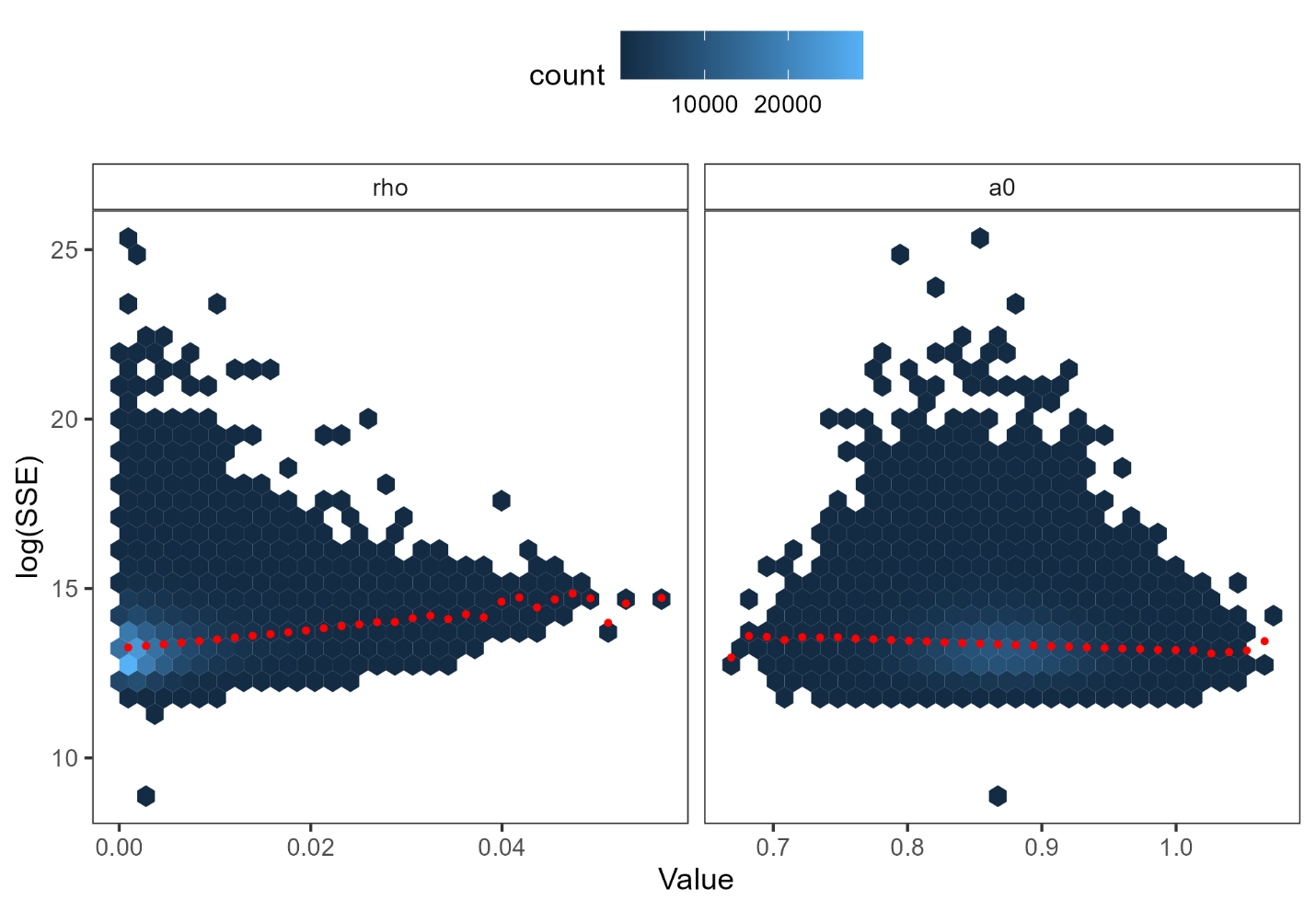


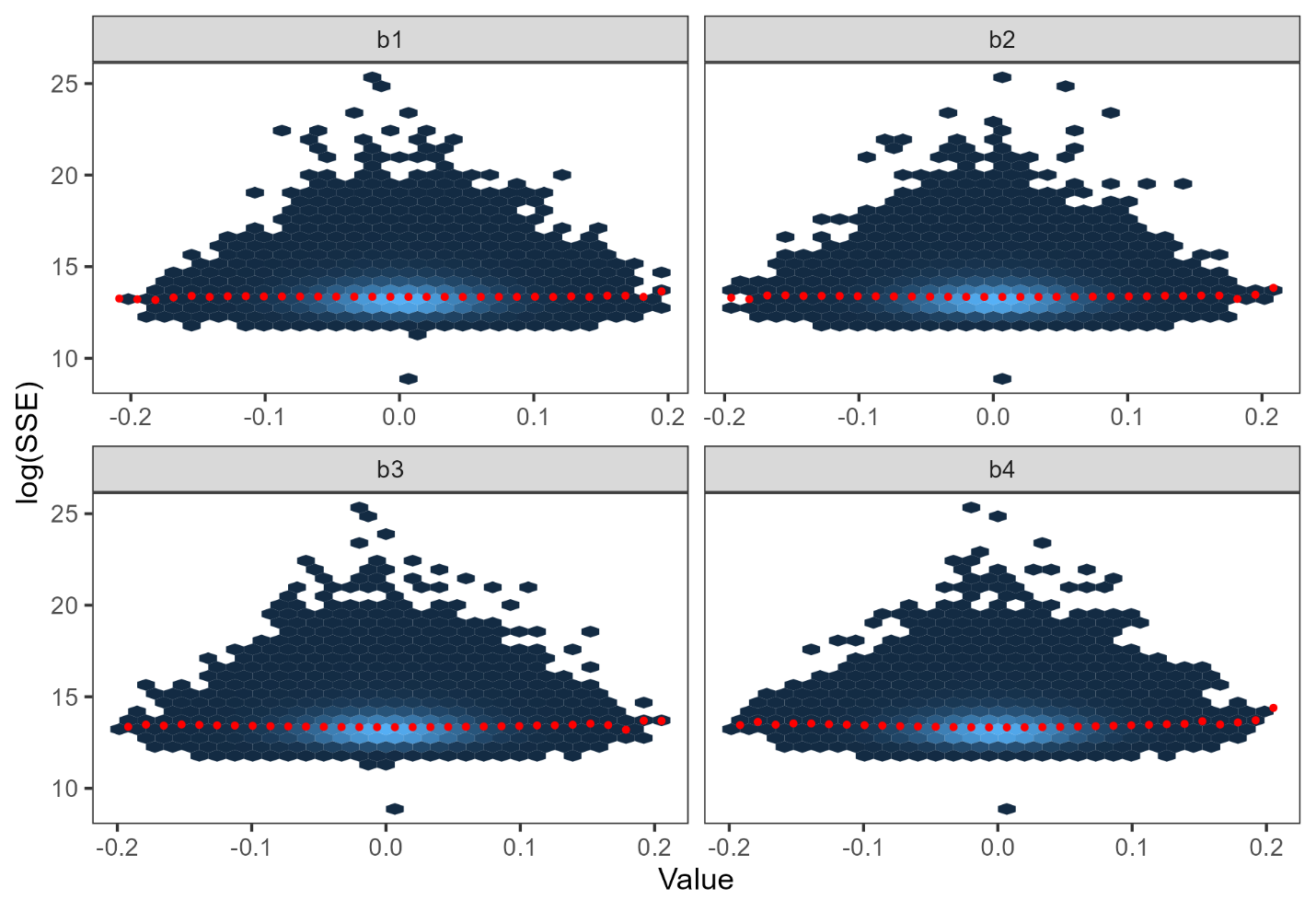

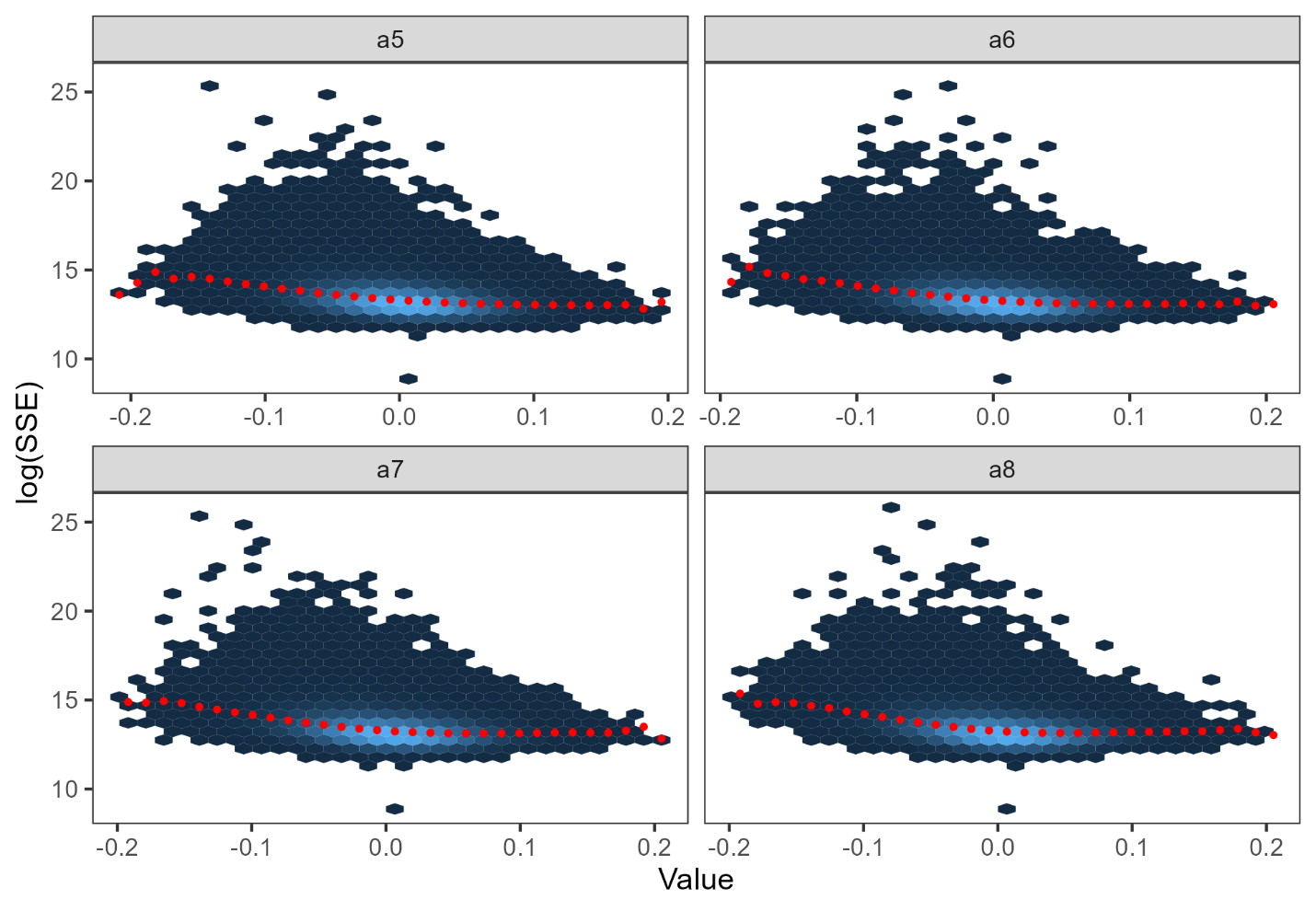


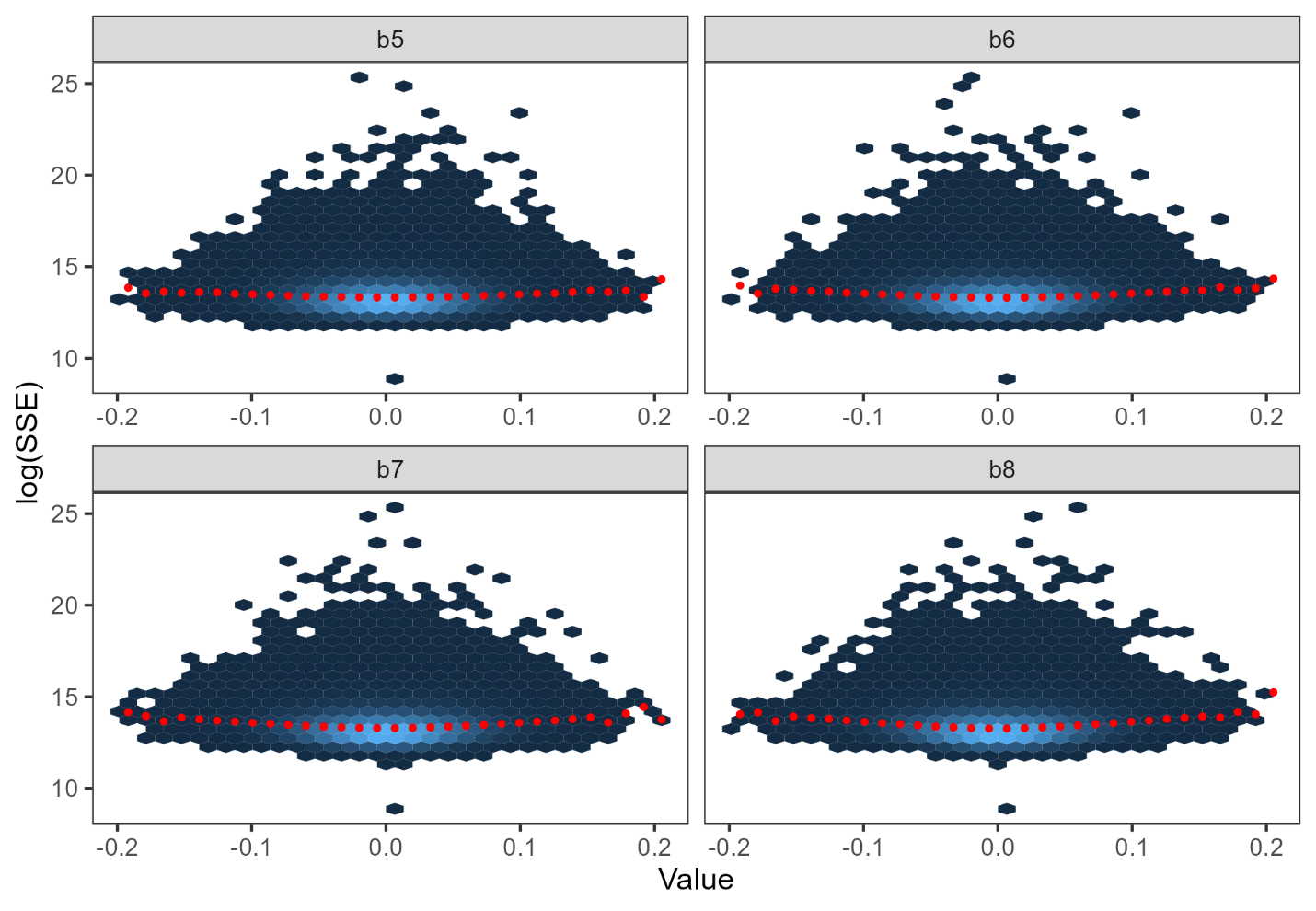


Figure S2. Scatter plots of model inputs against outputs for equation S2. The red dots show the mean of log(SSE), where the shade of the blue hexagons represent the count of the number of simulations within the hexagon bin.

Table S1. Parameters and their distributions for sensitivity analysis

| Parameter | Fitted value | Distribution |
| --- | --- | --- |
| $\rho$ | $4.467\times{10}^{-3}$ | $\mathrm{Exp}\left( 1/\rho\right)$ |
| $a_{0}$ | $8.70\times{10}^{-1}$ | $N\left( a_{0},\sigma_{ab}^{2} \right)$ |
| $a_{1}$ | $-3.86\times{10}^{-4}$ | $N\left( a_{1},\sigma_{ab}^{2} \right)$ |
| $a_{2}$ | $-5.34\times{10}^{-4}$ | $N\left( a_{2},\sigma_{ab}^{2} \right)$ |
| $a_{3}$ | $6.14\times{10}^{-5}$ | $N\left( a_{3},\sigma_{ab}^{2} \right)$ |
| $a_{4}$ | $1.43\times{10}^{-5}$ | $N\left( a_{4},\sigma_{ab}^{2} \right)$ |
| $a_{5}$ | $-9.15\times{10}^{-5}$ | $N\left( a_{5},\sigma_{ab}^{2} \right)$ |
| $a_{6}$ | $6.60\times{10}^{-5}$ | $N\left( a_{6},\sigma_{ab}^{2} \right)$ |
| $a_{7}$ | $7.93\times{10}^{-6}$ | $N\left( a_{7},\sigma_{ab}^{2} \right)$ |
| $a_{8}$ | $-6.64\times{10}^{-5}$ | $N\left( a_{8},\sigma_{ab}^{2} \right)$ |
| $b_{1}$ | $5.26\times{10}^{-4}$ | $N\left( b_{1},\sigma_{ab}^{2} \right)$ |
| $b_{2}$ | $8.33\times{10}^{-4}$ | $N\left( b_{2},\sigma_{ab}^{2} \right)$ |
| $b_{3}$ | $-4.78\times{10}^{-5}$ | $N\left( b_{3},\sigma_{ab}^{2} \right)$ |
| $b_{4}$ | $3.50\times{10}^{-4}$ | $N\left( b_{4},\sigma_{ab}^{2} \right)$ |
| $b_{5}$ | $1.70\times{10}^{-4}$ | $N\left( b_{5},\sigma_{ab}^{2} \right)$ |
| $b_{6}$ | $1.32\times{10}^{-4}$ | $N\left( b_{6},\sigma_{ab}^{2} \right)$ |
| $b_{7}$ | $-3.29\times{10}^{-5}$ | $N\left( b_{7},\sigma_{ab}^{2} \right)$ |
| $b_{8}$ | $3.01\times{10}^{-6}$ | $N\left( b_{8},\sigma_{ab}^{2} \right)$ |
